# Acid leaching process of an ultramafic mine tailing for indirect CO_2_ mineralization

**DOI:** 10.1038/s41598-026-35873-z

**Published:** 2026-03-14

**Authors:** Kyoung Hun Choi, Spencer Cunningham, Hamid Radfarnia, Kourosh Zanganeh, Gisele Azimi

**Affiliations:** 1 Laboratory for Strategic Materials, Department of Chemical Engineering and Applied Chemistry, University of Toronto, ON, M5S3E5, Toronto, 200 Collge St Canada; 2 Natural Resources Canada, CanmetENERGY-Ottawa, 1 Haanel Drive, ON, K1A1M19, Ottawa, Canada

**Keywords:** Sustainability, Carbon capture, Mineral carbonation, Ultramafic tailings, Mining, Acid leaching, Chemical engineering, Chemical engineering

## Abstract

The extraction of divalent metals from ultramafic mine tailings for indirect CO₂ mineralization is a promising strategy for large-scale carbon sequestration. This study investigates the acid leaching process of an ultramafic nickel tailing, rich in magnesium silicates, using two different acids: hydrochloric acid and citric acid. The aim was to evaluate the leaching efficiency of key metals, including magnesium, calcium, and iron, under varying operational conditions. These conditions included acid concentration (0.5 to 2 mol/L), solid-to-liquid (S/L) ratio (0.01 to 0.5 g/mL), temperature (25 to 65 °C), and multi-stage leaching. The experimental results show that both acids are effective in leaching the target metals, but with differing efficiencies. HCl, being a strong acid, exhibited higher leaching efficiency, particularly for Mg and Fe, due to its complete dissociation and greater ability to break down the mineral structure. Citric acid, a weaker organic acid, demonstrated more moderate leaching efficiency, especially for Mg, with a relatively lower impact from increased acid concentrations. The efficiency of metal extraction in both systems decreased with higher S/L ratios, likely due to reduced surface area for leaching, and the formation of more viscous slurries that hindered the movement of the leachant. Temperature had a distinct effect on the two acid systems. In the HCl system, increasing temperature resulted in a decrease in leaching efficiency for some elements like manganese, nickel, and sulfur, likely due to precipitation or changes in the solubility of certain metal species. In contrast, in the citric acid system, elevated temperatures improved the leaching efficiency for elements such as Mg, Fe, and Mn, enhancing the complexation and solubility of metals. However, temperature had minimal effect on other elements that do not readily form stable complexes with citric acid. The findings highlight that both HCl and citric acid can be effectively used for the acid leaching step in indirect CO₂ mineralization, with HCl providing higher extraction efficiencies for faster leaching and broader applicability to various metals, while citric acid offers a more environmentally friendly, less corrosive alternative. The results provide valuable insights into optimizing the leaching conditions to improve the overall efficiency of indirect mineral carbonation, which could contribute to more sustainable mining practices and enhanced CO₂ sequestration technologies.

**Supplementary Information:**

The online version contains supplementary material available at 10.1038/s41598-026-35873-z.

## Introduction

 Industrial greenhouse gas (GHG) emissions are a major driver of anthropogenic climate change. In 2023, emissions from primary mineral and metal production were estimated to contribute 7% of global energy-related GHG emissions^1^. In Canada, heavy industry, including mining, was responsible for 41% of the country’s total GHG emissions in 2022, amounting to 88.8 Mt of (carbon dioxide) CO_2_ equivalent (CO_2_e) emissions^2^. As part of a global effort to limit global warming to 2 °C, Canada pledged to reduce GHG emissions by 30% from 2005 levels (which is 58.62 Mt CO_2_ equivalent) under the 2015 Paris Agreement, which includes commitments from 197 other countries^3^. To meet the Paris Agreement, governments and industries are investing in innovative solutions to reduce GHG emissions.

Supplementary Figure S1a present the contribution of various sectors to CO_2_ emissions globally. Supplementary Figure S1b presents the share of major contributing countries^4^. As shown, power industry is the most contributing industry followed by transport, non-combustion, building and mining. Among the countries, China has the highest CO_2_ generation share followed by the United States, India, Russia, and Japan.

There are three primary strategies for reducing CO_2_ emissions: utilizing energy-efficient technologies to lower fossil fuel consumption, adopting renewable energy sources such as solar, wind, biomass, or nuclear, and implementing Carbon Capture and Storage (CCS) technology. CCS involves three main steps: separating CO_2_ from other flue gases, transporting the separated CO_2_ to a storage site, and permanently isolating CO_2_ from the atmosphere using various sequestration methods^5^.

The four main CO_2_ sequestration methods, each with distinct characteristics, limitations, longevity, and costs, are mineral, biological, geological, and ocean sequestration. Among these, mineral sequestration offers the highest CO_2_ storage capacity, effectively sequestering high percentage of CO_2_ emissions^4^. It is also considered safer compared with geological or ocean storage methods, which have associated environmental concerns^6,7^. Table 1 presents the advantages and drawbacks of the different methods of CO_2_ sequestration. The end products of mineral sequestration also have potential industrial applications^8^.

**Table 1:** Advantages and drawbacks of different CO_2_ sequestration methods.

Storage types	Benefits	Drawbacks	Gt of sequestered CO_2_	Cost $/t-CO_2_	Reference
Geological	Established technology Cost-effective Publicly accepted	High risk of leakage Requires continuous monitoring Lack of suitable sites	8,000–55,000	0.5–8.0	^9,10^
Ocean	High potential Widely available No need for monitoring	High environmental risk More expensive than geological storage Negative/hostile public perception	1,400 − 20,000	6–31	^11,12^
Biological	Low cost Minimal environmental impact High potential	Monoculture plantations can disrupt ecosystem balance Insufficient capacity Risk of disrupting the food chain	18.3	2–100	^13,14^
Mineral carbonation	Environmentally friendly Exothermic reactions Abundant feedstock Permanent solution No need for monitoring Utilization of industrial waste	High expenses Slow reaction rates Necessity for feedstock pre-treatment High energy requirements Uncertain future viability Inefficient recovery of additive chemicals Challenges in disposing of or utilizing the process byproducts on a large scale Environmental impact from feedstock mining	> 10,000 to 1,000,000	50–100	^15,16^

According to an article by McKinsey & Company^17^, mining companies can reduce GHG emissions through various strategies, such as enhancing energy efficiency in processes, transitioning to renewable energy sources, electrifying trucks and equipment, and using hydrogen fuel-powered engines. Another effective approach to mitigate GHG emissions in the mining sector is mineral carbonation, which captures and stores carbon dioxide on a large scale^4^.

Mineral carbonation is a natural process where common ultramafic minerals react with CO_2_ to form stable minerals and inert by-products, effectively sequestering CO_2_ within sediment minerals^18–20^. The resulting products from this process have been found to possess enhanced material properties, making them suitable for applications such as concrete additives or as cementitious materials^21^. Mining operations that extract valuable metals from ultramafic deposits generate large amounts of carbon-sequestering byproduct, offering significant potential for carbon capture and the repurposing of materials^18^.

Recent government legislations, such as the Canadian carbon credit system, offer financial incentives for industries to reduce their carbon footprint. Carbon credits provide an alternative revenue stream for companies based on the amount of carbon dioxide they capture. These incentives make ultramafic deposits more appealing for development due to their potential for carbon capture from mine tailings. Demonstrating carbon sequestration can not only generate additional revenue for mines but also enhance the environmental aspect of a deposit’s environmental, social, and governance (ESG) evaluation and improve the marketability of the metal product. Notable examples of carbon capture via mineral carbonation of tailings in mining sectors include the Baptiste Nickel Project (FPX Nickel Corp.), the Crawford Nickel Project (Canada Nickel Co.), and the Dumont Nickel Project (Magneto Investments LP)^18^.

Mineral carbonation reactions can be categorized into two categories: direct and indirect carbonation. In direct mineral carbonation, the carbonation and mineral dissolution processes happen simultaneously in a single step, resulting in the formation of a carbonate product. Conversely, indirect mineral carbonation involves a multi-step process where divalent metals are first extracted from feedstock minerals before undergoing carbonation in a separate step. Metals like Mg, Ca, or Fe are extracted under favorable conditions and typically precipitated as highly reactive hydroxides. These metal precipitates are then introduced into a carbonation reactor, where they form carbonate minerals under optimal alkaline and elevated CO_2_ conditions^22^.

A straightforward example of indirect mineral carbonation is acid extraction, which effectively extracts divalent metals from unreactive silicate minerals using various acids such as HCl, H_2_SO_4_, and HNO_3_. Acid extraction leverages the favorable mineral dissolution kinetics under acidic conditions, significantly enhancing the extraction of divalent metals from the minerals within a reasonable timeframe. This increased extraction efficiency facilitates a greater conversion of metal-bearing minerals into carbonate products^23^. Other indirect carbonation methods include alkaline rocks or wastes, such as serpentinites, fly ash, concrete sludge, steel slags, red gypsum, and phosphogypsum^24^.

Mineral carbonation in industrial applications is frequently studied using ultramafic minerals found in mine tailings^25,26^. Ultramafic and mafic rocks, which make up the majority of the Earth’s mantle, are rich in magnesium and iron, making them ideal candidates for mineral carbonation. Ultramafic rocks like olivine, pyroxene, and clinopyroxene have shown significant potential for CO_2_ sequestration^18^.

Ultramafic and mafic deposits are economically mined for valuable metals such as nickel, cobalt, and copper, constituting 7% of the total annual global metal value^27^. As high-grade sulfide ores become increasingly scarce, the nickel industry is showing growing interest in developing low-grade nickel deposits often found in ultramafic rocks^23^. After the extraction of valuable metals, large quantities of waste rock rich in magnesium silicates and small amounts of reactive brucite are stored in tailings storage facilities (TSFs) generally called tailing ponds. These TSFs offer significant potential for large-scale mineral carbonation, even under suboptimal reaction conditions^25^.

In this study, we investigated the acid leaching process of an ultramafic nickel tailing containing brucite to prepare a solution for indirect mineral carbonation. Both hydrochloric acid (inorganic) and citric acid (organic) were used as leaching agents. The selection of these two acids was based a preliminary thermodynamic simulation of the solution using OLI software (v. 12.0). We examined the impact of various factors, including acid concentration, solid-to-liquid ratio, temperature, and multi-stage leaching on the leaching efficiency of elements such as magnesium, calcium, and iron. Samples before and after leaching were characterized using X-ray diffraction, electron probe micro analyzer, and scanning electron microscopy energy dispersive spectroscopy to elucidate the extraction mechanism.

While most previous studies have concentrated on the CO_2_ capture step of indirect mineral carbonation, research on the leaching step has been limited. To the best of our knowledge, no prior study has reported the acid leaching mechanism of ultramafic nickel tailings using both hydrochloric acid and citric acid. This study aims to fill this knowledge gap. The findings will provide a better understanding of the leaching mechanisms and improve the overall efficiency of the indirect mineral carbonation process. Future research will focus on utilizing the leachate produced in this study for CO_2_ capture to evaluate its capacity, potentially leading to more effective carbon sequestration methods.

The present study focuses exclusively on the acid leaching stage of the indirect CO₂ mineralization process. Acid leaching serves as the first and rate-limiting step of the pH-swing route, where divalent metal ions (Mg²⁺, Ca²⁺, Fe²⁺/Fe³⁺) are extracted into solution and subsequently used for carbonate precipitation under alkaline and CO₂-rich conditions. Because the carbonation step involves different reaction mechanisms, mass-transfer limitations, and kinetic behavior, its evaluation is presented in a separate dedicated study^28^. That companion work uses the leachates generated to quantify CO₂ sequestration capacity, analyze mineralogical changes during carbonation, and examine process intensification strategies. Together, the two studies provide a complete assessment of the indirect mineral carbonation workflow.

The purpose of this study is to investigate the fundamental dissolution behavior of Mg-bearing ultramafic tailings as the initial step in the indirect CO₂ mineralization process. Although direct acid leaching of tailings is not currently practiced for economic gain, these materials are generated in large volumes and stored on site, making them readily available for carbon-removal applications. Their use in CO₂ mineralization may provide economic value through carbon-credit generation, reduction of waste-management liabilities, and potential co-recovery of critical metals (e.g., Ni, Co, Fe) from the leachate. Therefore, the leaching results presented here should be interpreted as providing the mechanistic basis for evaluating process feasibility rather than as a stand-alone commercial leaching route. A full technoeconomic analysis is beyond the scope of the present study but can be conducted in future work using the dissolution data reported here.

## Results and discussion

### Characterization of the Ni tailing

#### Alkali fusion followed by ICP-OES

The elemental analysis of the sample was performed on three samples, and the mean results along with the relative standard deviation (RSD) are summarized in Table 2. The sample contains 231.14 g/kg of Mg, 13.99 g/kg of Al, 25.90 g/kg of Fe, and 182.93 g/kg of Si.

**Table 2:** Chemical composition of the feed Ni tailing.

Element	Mg	Ca	Mn	Al	Fe	Ni	Cr	S	Si
Mean (g/kg)	231.14	7.30	0.62	13.99	25.90	1.16	3.39	3.14	182.93
RSD (%)	3.5	0.3	0.3	0.4	0.6	0.2	0.3	0.1	3.1

#### X-Ray diffraction

**Fig. 1:**
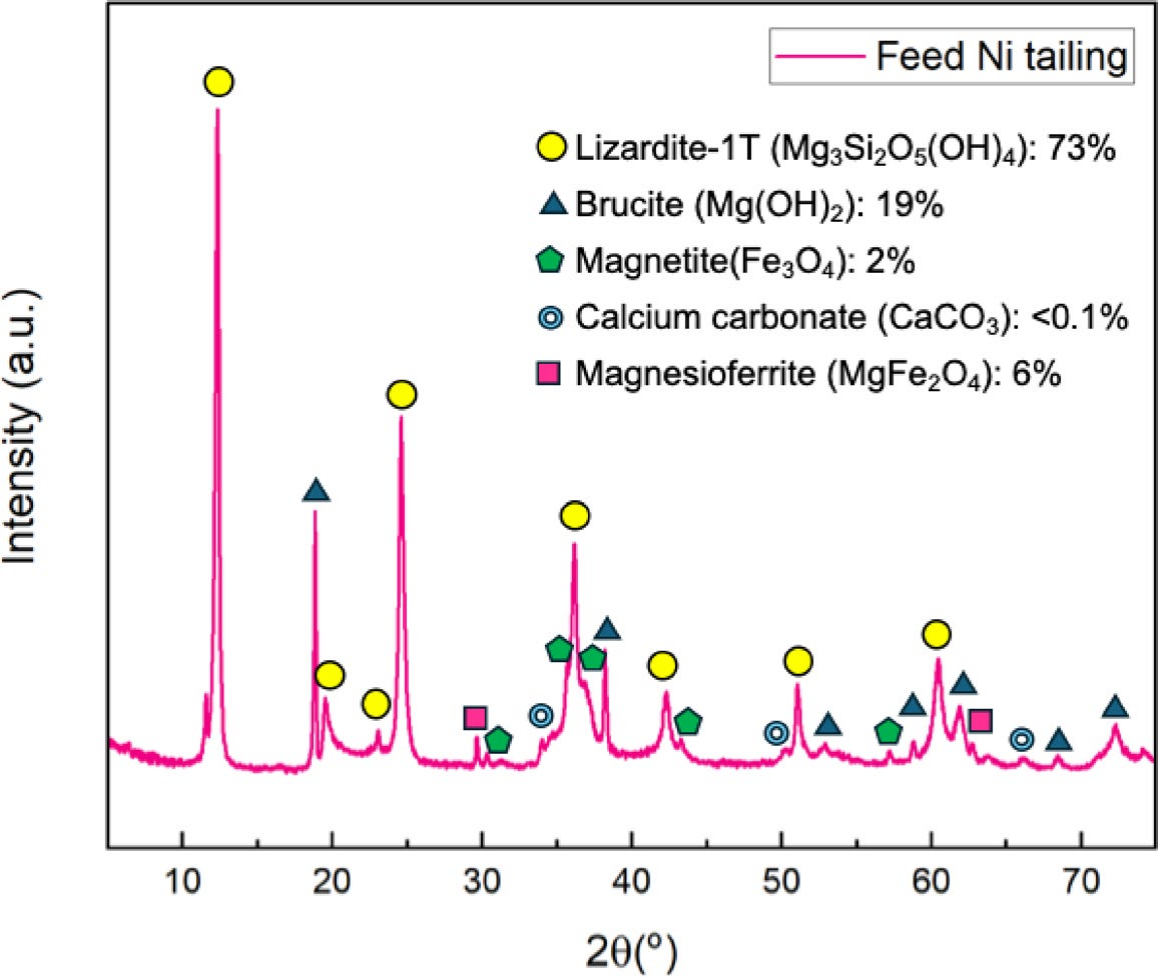
XRD patten of the Ni tailing feed. The Rietveld analysis results are also presented.

The mineralogical characteristics of the Ni tailing sample obtained from X-ray diffraction analysis are presented in Fig. 1, indicating the sample mainly contains lizardite (Mg_3_Si_2_O_5_(OH)_4_), brucite (Mg(OH)_2_), and magnetite (Fe_3_O_4_). Based on the Rietveld analysis, the sample contains 73% lizardiate, 2% magnetite, 19% brucite, and 6% magnesioferrite (MgFe_2_O_4_). A very small amount of calcium carbonate is also detected.

#### SEM-EDX results

**Fig. 2:**
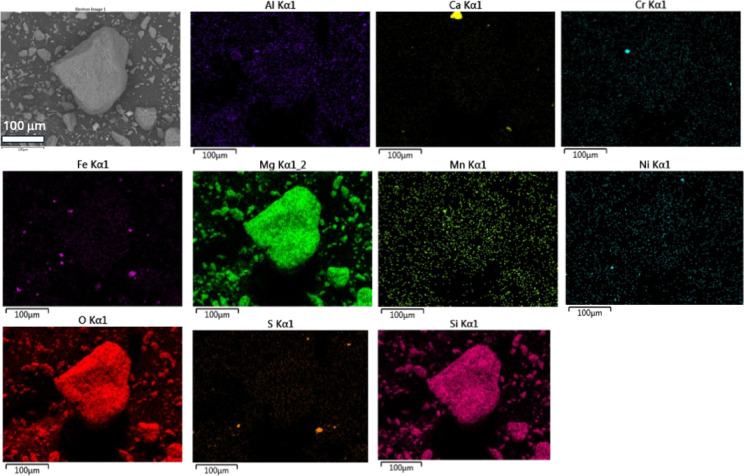
Backscattered electron (BSE) image and elemental mapping of the feed Ni tailing sample.

Elemental mapping of the feed, as illustrated in Fig. 2, revealed a predominance of grains comprising Mg, Si, and O (Lizardite). Other grains containing Mg, Fe, O and grains containing Fe and O are detected. Backscattered secondary electron (BSE) image in Supplementary Figure S2 indicates the sample particle morphology, texture, and size. As shown, particles have a relatively smooth surface, and a dense texture is observed.

### Kinetic experiment results

To determine the equilibration time in both HCl and citric acid systems, two kinetic tests were conducted over a 6-h period, with samples taken at various intervals. The concentration of both acids was selected at 1 mol L^− 1^, at a temperature of 25 °C and a S/L ratio of 0.25 g mL^− 1^. Figures S3a-c display the concentration profiles of Mg, Ca, and Ni in the HCl and citric acid systems, respectively. The results indicated that the equilibration times for HCl and citric acid were 180 min and 300 min, respectively. These equilibration times were subsequently used as the residence times for the respective systems in the other investigations throughout this study.

### Effect of operating parameters

#### Effect of S/L ratio

**Fig. 3:**
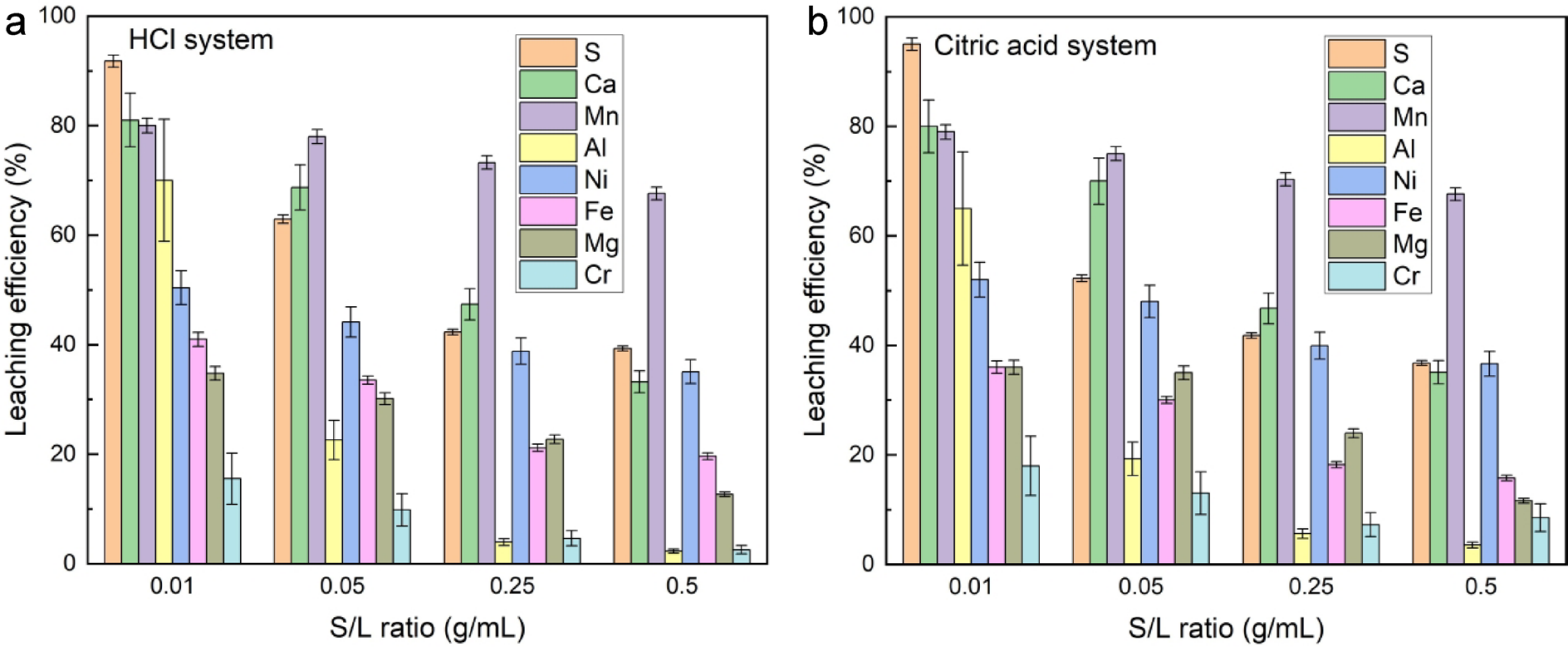
Effect of S/L ratio on elements’ leaching efficiency in (**a**) HCl, (**b**) citric acid at 25 °C, 1 mol L^-1^ acid concentration and agitation rate of 400 rpm with 180- and 300-min residence time, respectively. The error bars are determined based on three replicates.

The effect of the S/L ratio was investigated in both HCl and citric acid systems. The acid concentration was maintained at 1 mol L^− 1^, with a temperature of 25 °C, agitation at 400 rpm, and residence times of 180 min for HCl and 300 min for citric acid. The S/L ratios tested were 0.01, 0.05, 0.25, and 0.5 g mL^− 1^. As shown in Fig. 3a, in the HCl system, the leaching efficiency decreased with increasing S/L ratio. Similarly, Fig. 3b shows that in the citric acid system, leaching efficiency also decreased with increasing S/L ratio. The leaching efficiency of all elements were similar in both systems.

The decrease in leaching efficiency with increasing S/L ratio can be explained by several factors including reduced surface area for leaching, limited solution volume (reaching saturation limit), increased viscosity, and precipitation or saturation of leachant. As the S/L ratio increases, there is less liquid available to dissolve the metals. As the solid-to-liquid ratio increases, the higher concentration of nonmetallic materials promotes the agglomeration of raw materials. This leads to more frequent collisions and friction between the particles during the leaching process, hindering the thorough contact between metals and the leaching solution, which in turn reduces leaching efficiency^29^. Also, at higher S/L ratios, there is less leachant (acid) to dissolve the metals. The concentration of the leaching agent becomes less concentrated, which can reduce its ability to effectively break down the minerals in the tailings and extract the divalent metals. Additionally, higher S/L ratios can lead to a more viscous slurry, which may hinder the movement of the leachant throughout the sample, reducing its ability to interact with the solid particles and extract the metals. Finally, with more solid material, the leaching solution may become saturated with dissolved metals, leading to precipitation or a reduced ability to keep metals in solution, which limits further leaching. These factors combined can lead to a decrease in the leaching efficiency as the S/L ratio increases.

#### Effect of acid concentration

**Fig. 4:**
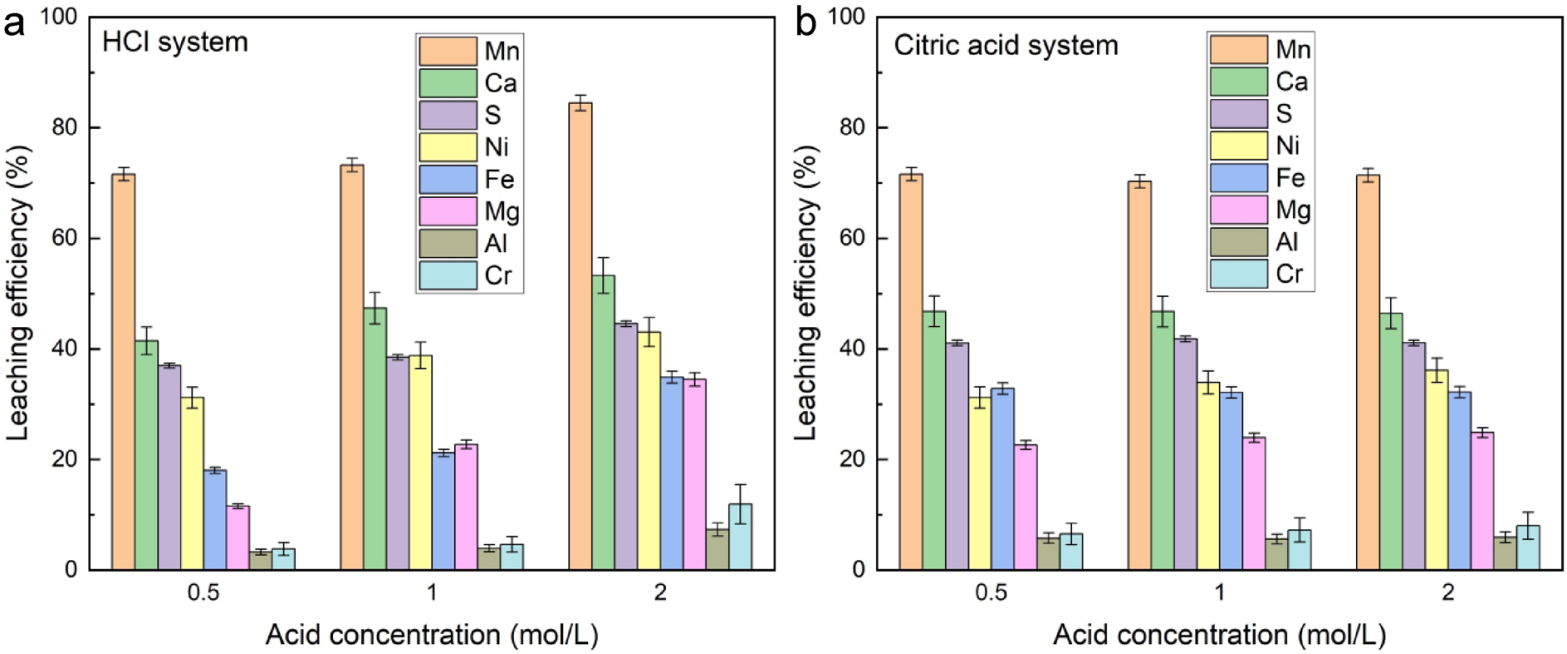
Effect of acid concentration on elements’ leaching efficiency in (**a**) HCl, (**b**) citric acid at 25 °C, S/L ratio of 0.25 g mL-1 and agitation rate of 400 rpm with 180- and 300-min residence time, respectively. The error bars are determined based on three replicates.

The effect of acid concentration was examined in both HCl and citric acid systems. The S/L ratio was maintained at 0.25 g mL^− 1^, the temperature at 25 °C, agitation at 400 rpm, and residence times of 180 min for HCl and 300 min for citric acid. The concentrations tested were 0.5, 1, and 2 mol L^− 1^. As shown in Fig. 4a, in the HCl system, leaching efficiency increased with higher acid concentrations. Conversely, as shown in Fig. 4b, in the citric acid system, leaching efficiency remained almost unchanged with increasing acid concentration.

The different effects of acid concentration on leaching efficiency for HCl and citric acid can be attributed to the distinct chemical behaviors and mechanisms of these acids. HCl is a strong acid (K_a_=1.3$\:\times\:$10^6^) that dissociates completely in solution, providing a high concentration of hydronium ions. These hydronium ions are effective at breaking down the mineral structure of the tailings and facilitating the dissolution of metals. As the concentration of HCl increases, the availability of hydronium ions increases, which improves the leaching efficiency. Higher acid concentrations can more effectively break down the minerals, thus enhancing the metal extraction process.

In contrast, citric acid is a weak acid (K_a1_=7.1$\:\times\:$10^−4^) that does not dissociate as fully as HCl. While citric acid can form complexes with metals, the rate of dissociation and the availability of hydronium ions is much lower than in strong acids like HCl. Citric acid primarily leaches metals by forming metal-ligand complexes rather than through protonation. At higher concentrations, the ability of citric acid to form these complexes may not increase significantly beyond a certain point. Once the citric acid is sufficiently concentrated to form complexes with the metals, further increases in concentration have a reduced effect on the leaching efficiency. In some cases, increasing the citric acid concentration further may not provide additional benefits because the active sites on the metals for complex formation become saturated, leading to diminishing returns in metal leaching.

#### Effect of temperature

**Fig. 5:**
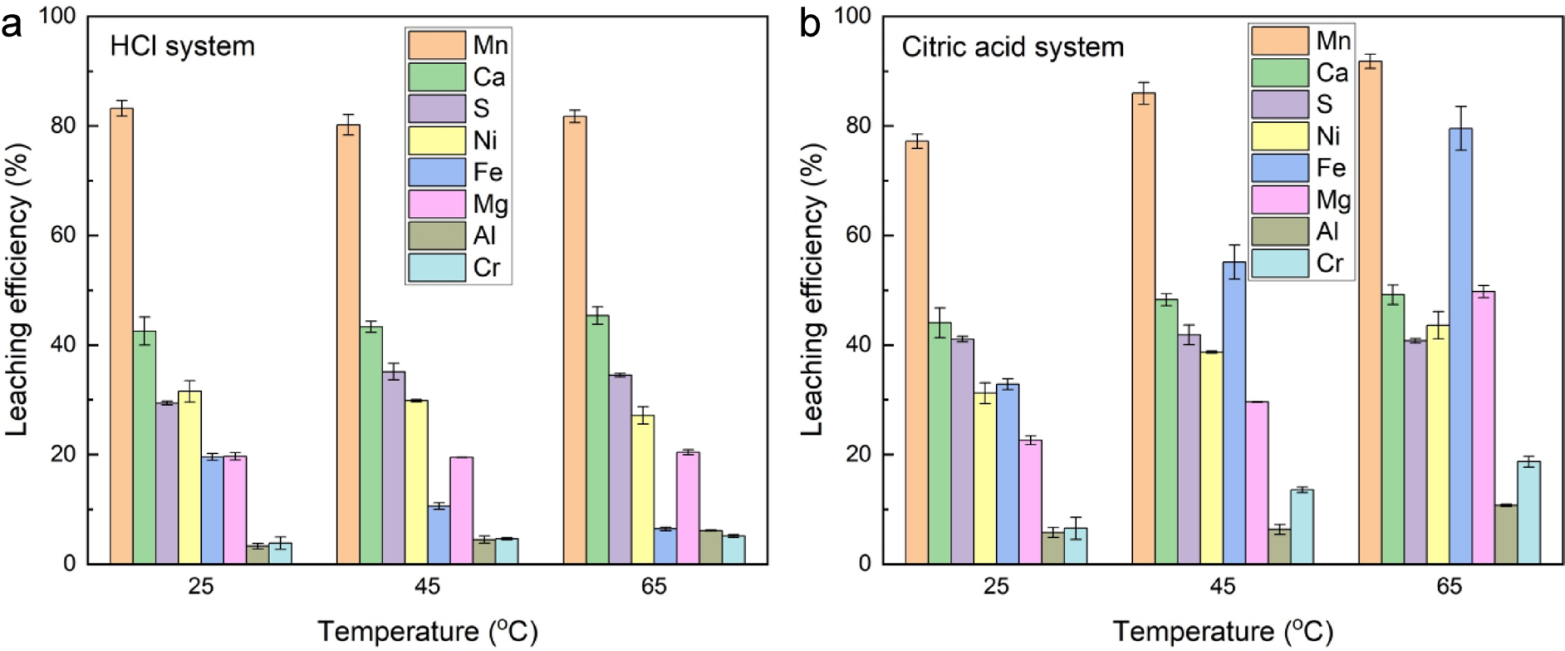
Effect of temperature on elements’ leaching efficiency in **a**) HCl, **b**) citric acid at 1 mol L-1 acid concentration, S/L ratio of 0.25 g mL^- 1^ and agitation rate of 400 rpm with 180- and 300-min residence time, respectively. The error bars are determined based on three replicates.

The effect of temperature was examined in both HCl and citric acid systems. The S/L ratio was maintained at 0.25 g mL^− 1^, with an acid concentration of 1 mol L^− 1^, agitation at 400 rpm, and residence times of 180 min for HCl and 300 min for citric acid. The temperatures tested were 25, 45, and 65 °C. As illustrated in Fig. 5a, in the HCl system, the leaching efficiency for some elements, such as Mn, Ni, and S, decreased with increasing temperature, while it remained almost constant for other elements. Conversely, as shown in Fig. 5b, in the citric acid system, the leaching efficiency for elements like Mn, Fe, Ni, Cr, and Mg increased with rising temperature, while it remained nearly constant for others.

The contrasting effects of temperature on leaching efficiency in the HCl and citric acid systems can be attributed to differences in the leaching mechanisms, the nature of the acids, and the behavior of the elements involved in the leaching process. In the HCl system, increasing temperature typically enhances the solubility of metals by increasing the kinetic energy of both the acid and metal ions. However, for some elements like Mn, Ni, and S, an increase in temperature may lead to precipitation or changes in the speciation of the metal ions. At higher temperatures, some elements might undergo precipitation reactions, particularly if the metal forms insoluble compounds or if the solubility limits of certain metal ions are exceeded^30^. This could explain why elements like Mn, Ni, and S show decreased leaching efficiency at higher temperatures. Additionally, HCl, being a strong acid, dissociates fully, and its efficiency in breaking down the mineral structure is generally not strongly influenced by temperature. The dissociation of HCl in water is exothermic. According to Le Chatelier’s principle, increasing temperature slightly shifts the equilibrium backward (favoring the undissociated form), but this effect is negligible in small temperature variations^31^.

Citric acid works by forming metal-ligand complexes rather than relying solely on protonation like HCl. As the temperature rises, the chelation process may become more efficient for elements like Mn, Fe, Ni, Cr and Mg, as higher temperatures can enhance the kinetics of the complexation reactions. As temperature increases, the kinetic energy of molecules rises, potentially enhancing the ability of citric acid to form stable complexes with metals, leading to improved leaching efficiency for metals like Mn, Fe, and Mg. The increased temperature may also improve the solubility of certain metals in the citric acid solution, allowing for greater extraction. For example, Fe forms strong complexes with citric acid, especially Fe³⁺. Solubility increases significantly with temperature due to both complexation and temperature effects. In the case of Fe²⁺, oxidation to Fe³⁺ can also occur at higher temperatures, affecting solubility^32^. Mn²⁺ forms moderately strong complexes with citric acid. Solubility increases with temperature, but less dramatically than Fe. Oxidation to Mn³⁺ or Mn⁴⁺ at higher temperatures can lead to precipitation as oxides, slightly limiting solubility under oxidative conditions^32^. Mg forms weaker complexes with citric acid compared with Fe and Mn. Solubility still increases with temperature, but primarily due to the endothermic nature of the dissolution process rather than complexation strength^32^. Cr³⁺ forms strong complexes with citric acid, enhancing its solubility. The solubility of Cr³⁺ increases with temperature due to the endothermic nature of both dissolution and complexation reactions. Cr⁶⁺ solubility is less dependent on citric acid, as it exists as highly soluble anions (CrO₄²⁻, HCrO₄⁻), but complexation with citric acid is weak^32^. Ni²⁺ forms moderate-strength complexes with citric acid. Solubility increases with temperature due to endothermic dissolution and complexation. At higher temperatures, complex stability increases, improving solubility^32^. Ca²⁺ forms weak complexes with citric acid compared with transition metals. Solubility increases moderately with temperature, primarily due to the endothermic dissolution of calcium salts. Unlike other metals, the effect of complexation with citric acid is less pronounced, but it still prevents precipitation as CaCO₃ in some conditions^32^.

The solubility of metal complexes with citric acid depends on several factors, including (1) complex stability constants (Kₛ): higher stability usually leads to higher solubility, (2) charge density of the metal ion: higher charge density promotes stronger complexation, (3) ionic radius: smaller ions with higher charges often form stronger complexes, and (4) hydrolysis tendencies: metals prone to hydrolysis may form insoluble hydroxides unless stabilized by complexation.

**Fig. 6:**
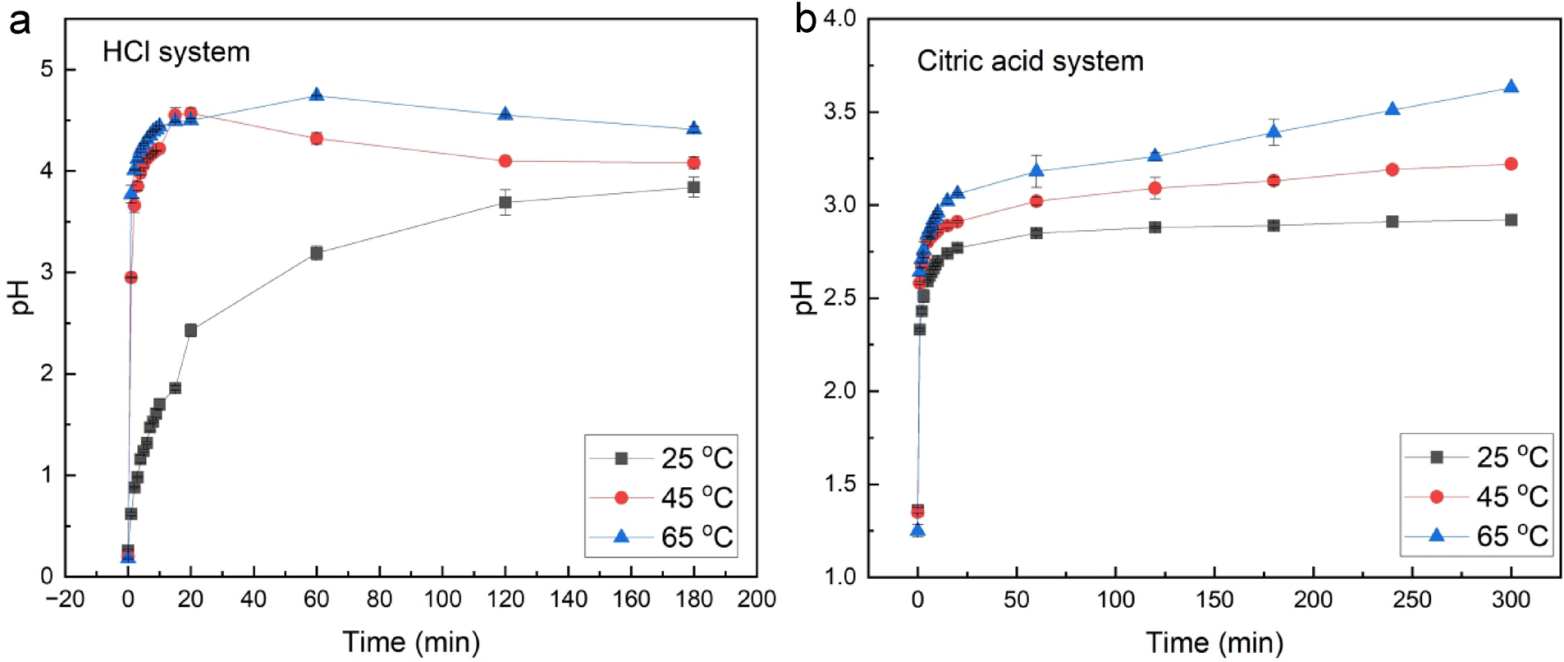
Effect of temperature on pH in **a**) HCl, **b**) citric acid at 1 mol L^- 1^ acid concentration, S/L ratio of 0.25 g mL^- 1^ and agitation rate of 400 rpm during 180- and 300-min residence time, respectively. The error bars are determined based on three replicates.

In this set of tests, the pH of the solution was measured over time, and the results are presented in Fig. 6a and b. Initially, the pH in the HCl system was approximately 0.2, increasing to around 3.8, 4.1, and 4.4 at 25, 45, and 65 °C after 180 min, respectively. In the citric acid system, the initial pH was about 1.3, rising to approximately 2.9, 3.2, and 3.6 at 25, 45, and 65 °C after 300 min.

The observed changes in pH over time in both the HCl and citric acid systems can be explained by the nature of the leaching processes, including the consumption of acid, formation of metal species, and the neutralization or buffering reactions that take place during the extraction of metals. Neutralization phenomenon as a result of dissolution of Ni-tailing constituents specifically brucite has a significant effect. Brucite is a main provider of hydroxyls during dissolution, producing MgCl_2_ which would result in acid consumption.

HCl is a strong acid that dissociates completely in solution, releasing hydronium ions that contribute to a low initial pH (around 0.2). During leaching, the acid reacts with the minerals in the tailings, dissolving metals and forming soluble metal ions or compounds. As the reaction progresses, the acid is consumed in the leaching process, primarily by the dissolution of metals. For example, the leaching of base metals like Ni or Mn results in the consumption of HCl, as it reacts with the mineral surface to form soluble metal chlorides. The consumption of acid leads to an increase in pH over time.

As the temperature increases, certain metal ions or metal species may precipitate or undergo changes in solubility. This can further affect the pH as the system moves toward equilibrium, leading to a rise in pH as the acid is consumed and the amount of free hydronium ions decreases. The increase in temperature (from 25 to 65 °C) can accelerate these reactions, causing a faster consumption of acid and a more significant increase in pH. This is why the pH increases more at higher temperatures (from 3.8 at 25 °C to 4.4 at 65 °C after 180 min).

Citric acid is a weak acid, meaning it does not dissociate completely in solution. At the start, the pH is higher (around 1.3) compared with the HCl system because the concentration of hydronium ions from citric acid is lower, given that it only partially dissociates.

As the leaching process continues, citric acid reacts with metals in the tailings to form metal-citrate complexes. This process consumes citric acid, but to a lesser extent than in the HCl system because citric acid is weaker, and the chelation reaction is slower and less aggressive than protonation-based dissolution. Also, citric acid can act as a buffer in the system, especially in the presence of metal ions, which may lead to a slower increase in pH. As the citric acid concentration decreases due to complex formation, the pH rises more gradually. The buffering capacity of citric acid helps stabilize the pH over time, but still, as acid is consumed, the pH increases. As with the HCl system, increasing temperature (from 25 to 65 °C) increases the reaction rate, leading to a faster consumption of acid and a more rapid rise in pH. At higher temperatures, the chelation process between citric acid and metals becomes more efficient, further consuming the acid and increasing the pH.

#### Effect of multi-stage leaching

**Fig. 7:**
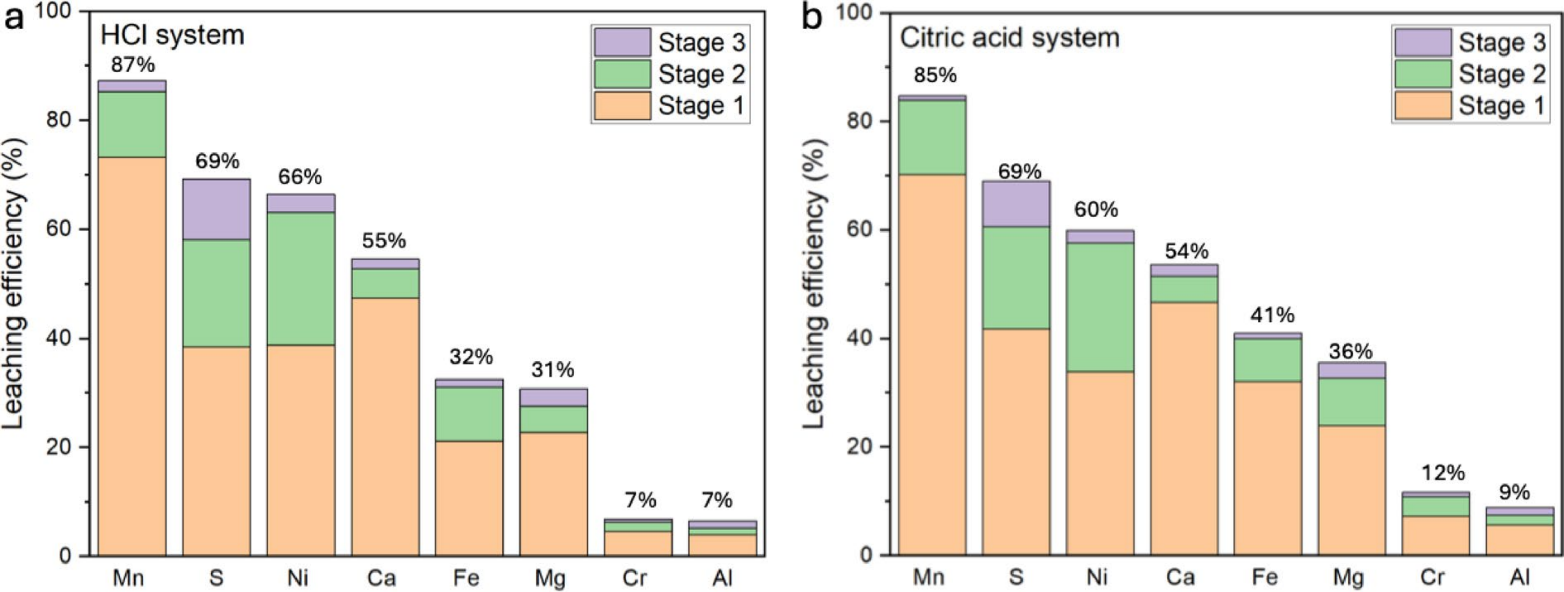
Effect of multistage leaching on the leaching efficiency of elements in (**a**) HCl, (**b**) citric acid at 1 mol/L acid concentration, 25 °C, S/L ratio of 0.25 g mL-1 and agitation rate of 400 rpm during 180- and 300-min residence time, respectively.

The effect of multi-stage leaching was examined in both HCl and citric acid systems. The parameters were set as follows: a S/L ratio of 0.25 g mL^− 1^, an acid concentration of 1 mol L^− 1^, agitation at 400 rpm, a temperature of 25 °C, and residence times of 180 min for HCl and 300 min for citric acid. In this process, the filter cake from the first stage was subjected to fresh solution in stages 2 and 3. Figure 7a and b show the results of multi-stage leaching on the leaching efficiency of target elements in HCl and citric acid, respectively. As depicted, some degree of extraction was observed for all elements at each stage. Maximum leaching efficiency was achieved in stage 1, followed by stage 2 and stage 3. The leaching efficiency in stage 3 was quite low, below 3% in most cases. In the HCl system, the Mg leaching efficiency was 23% in stage 1 and decreased to 5% and 3% in stages 2 and 3, respectively. In the citric acid system, the Mg leaching efficiency was 24% in stage 1 and decreased to 9% and 3% in stages 2 and 3, respectively.

In multi-stage leaching processes, the first stage typically achieves the highest leaching efficiency because most of the easily soluble metal content is extracted. By the second and third stages, the remaining metal content is more difficult to leach due to acid consumption, changes in the mineralogy of the remaining material, and possible formation of passivating layers for example SiO_2_^33^. This leads to a significant decrease in leaching efficiency in the later stages, which is particularly noticeable for Mg (and other metals), as shown by the lower extraction rates in stages 2 and 3 in both the HCl and citric acid systems.

Comparing the total leaching efficiency of all elements after three leaching stages in both HCl and citric acid systems indicates that the leaching efficiency of all elements is comparable between the two systems. Therefore, both acids are suitable for the acid leaching step, and the choice between them should be based on process economics and environmental impact. Table 3 compares the advantages and disadvantages of these two acids. HCl is more suitable for processes requiring high leaching efficiency, fast extraction, and the ability to dissolve a broad range of metals. However, it comes with higher environmental concerns and operational costs due to corrosion and reagent consumption. Citric acid, while less effective and slower for certain metals, offers a more environmentally friendly and less corrosive alternative. It may be preferred where lower environmental impact, safety, and cost considerations are more important, or where selective leaching is desired. The choice between HCl and citric acid should ultimately depend on the specific goals of the leaching process, including leaching speed, metal target elements, environmental considerations, and economic factors such as reagent and equipment costs.

**Table 3:** Comparison between HCl and citric acid.

Property	HCl	Citric Acid
Acid Strength	Strong (complete dissociation)	Weak (partial dissociation)
Leaching Efficiency	High, especially for base metals like Ni, Mn	Moderate, effective for some metals (e.g., Mg, Fe)
Leaching Kinetics	Faster leaching due to high acidity	Slower leaching, especially for less soluble metals
Environmental Impact	Higher, requires careful handling and disposal	Lower, biodegradable and safer to handle
Corrosivity	High, requires corrosion-resistant materials	Low, less corrosion to equipment
Selectivity	Less selective, effective for a wide range of metals	Selective for metals that form stable complexes
Suitability for Multi-Stage Leaching	Effective but may require more frequent replenishment	Suitable, especially for more gentle extraction

Although citric acid is an organic compound, its environmental impact is substantially lower than that of many alternative organic acids. Citric acid is a naturally occurring, biodegradable weak acid that is widely used in food, pharmaceutical, and environmental applications^34^. In the context of indirect mineral carbonation, the leachate containing citrate species is not discharged; instead, it is carried forward into the carbonation step, where a significant portion of the citric acid is consumed through metal–citrate complexation and subsequent pH adjustment. Following carbonation, the remaining organic species can be recovered or treated using established degradation pathways (e.g., aerobic/anaerobic biological oxidation, advanced oxidation processes, or neutralization with Ca²⁺ to promote citrate precipitation). As such, the use of citric acid does not pose a direct environmental release risk within the closed-loop system investigated in this study.

### Mechanistic investigation of the leaching process

To elucidate the mechanism behind the leaching process in both systems, the residue after the leaching step at 1 mol L^− 1^ acid concentration, 25 °C, S/L ratio of 0.25 g mL^− 1^ and agitation rate of 400 rpm during 180- and 300-min residence time for HCl and citric acid were, respectively, characterized with XRD, SEM-EDS, and EMPA.

The elemental composition of the leaching solutions obtained under the optimum leaching conditions is provided in Table 4. These data quantify the concentrations of major dissolved species (Mg, Ni, Ca, Fe, Si, etc.) and directly reflect the extent of dissolution achieved in each acid system.

Bulk chemical analysis of the solid residues could not be performed because the samples were no longer available for further testing. Nevertheless, the combination of XRD, BSE imaging, and EPMA elemental mapping provides detailed insight into the mineralogical and microchemical changes occurring during leaching. These analyses confirm the selective dissolution of brucite and partial decomposition of Fe-bearing phases, as well as the persistence of lizardite in both acid systems.

Because these mineralogical datasets document the identity, abundance, and transformation of the phases present before and after leaching, they provide equivalent mechanistic information to bulk residue composition and allow the dissolution pathways to be confidently interpreted.

**Table 4:** Elemental composition of the leaching solutions obtained under the optimum leaching conditions (1 mol L^- 1^ acid concentration, 25 °C, S/L ratio of 0.25 g mL-1 and agitation rate of 400 rpm during 180- and 300-min residence time for HCl and citric acid).

	mg/L							
	Mg	Ca	Mn	Al	Fe	Ni	Cr	S
HCl	11344.88	776.90	126.95	150.08	1128.16	148.87	38.26	457.45
Citric acid	12469.84	804.28	118.78	145.31	1892.01	105.75	74.86	501.17

**Fig. 8:**
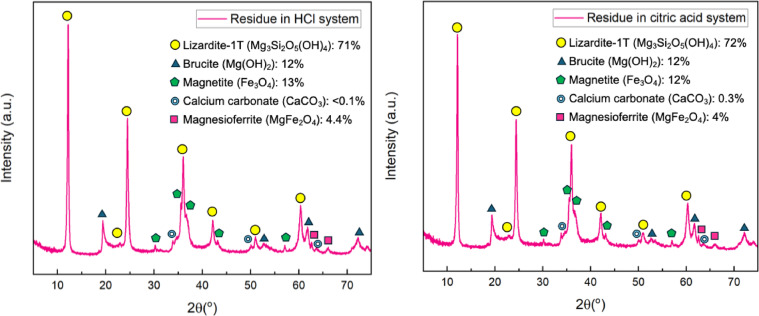
X-ray diffractograms of the leaching residue in (**a**) HCl, (**b**) citric acid at 1 mol L^- 1^ acid concentration, 25 °C, S/L ratio of 0.25 g mL^- 1^ and agitation rate of 400 rpm during 180- and 300-min residence time, respectively.

Figure 8a and b present the X-ray diffractograms of the residue in HCl and citric acid systems, respectively. Comparing these XRD graphs with one for the feed (Fig. 1) indicates that lizardite remained almost unchanged after leaching with both acids. Brucite decreased from 19% to 12% indicating its partial dissolution in both systems. The amount of magnetite increased from 2% to 12%, while magnesioferrite decreased from 6% to 4%.

The observations from the XRD results provide valuable insights into the leaching mechanism in both the HCl and citric acid systems. Here’s a proposed mechanism based on the mineralogical changes observed in the residue after leaching:

**(1) Stability of Lizardite**: Lizardite (a type of serpentine mineral) remained largely unchanged after leaching with both acids. This indicates that lizardite is relatively resistant to dissolution under the conditions tested (1 mol/L acid concentration, 25 °C, and the given S/L ratio). The proposed mechanism is as follows: Lizardite is a magnesium silicate mineral, and its dissolution typically requires stronger conditions or more aggressive reagents. In both HCl and citric acid systems, the conditions (particularly the acid concentration) may not have been sufficient to break down the silicate network, preventing its dissolution^35^.
**(2) Partial Dissolution of Brucite**: Brucite (magnesium hydroxide) decreased from 19% to 12% in both acid systems, indicating that partial dissolution occurred. The proposed mechanism is as follows: Both HCl and citric acid are capable of dissolving brucite. Brucite is a hydroxide mineral, and strong acids like HCl would protonate the hydroxide ions (OH⁻), leading to the dissolution of magnesium ions (Mg²⁺) into solution. Citric acid, though weaker, can also dissolve brucite through protonation and formation of magnesium-citrate complexes. The decrease in brucite could also be due to the formation of insoluble salts or complexes, causing a shift in the mineralogy but not complete dissolution.
**(3) Increase in Magnetite**: The increase in magnetite could be a result of the reduction of iron from other iron-containing minerals, such as magnesioferrite. In acidic environments, some ferric ions (Fe³⁺) may be reduced to ferrous ions (Fe²⁺), which can then recombine to form magnetite. This reduction could be promoted by the acidic conditions (in both HCl and citric acid systems) that facilitate the dissolution of iron minerals, followed by the re-precipitation of magnetite^36^.
**(4) Decrease in Magnesioferrite**: The decrease in magnesioferrite can be attributed to the dissolution of iron and magnesium from this mineral. In an acidic medium, magnesioferrite may undergo acid-induced dissolution, where the iron and magnesium components are leached into the solution. This would reduce the amount of magnesioferrite in the residue. The dissolved metal ions could either remain in solution or form new compounds (such as magnetite), as described above.

These observations imply that the leaching process is primarily dissolving more easily leachable minerals such as brucite, while iron-bearing minerals like magnesioferrite are undergoing reduction and transformation, leading to the formation of magnetite. The relative stability of lizardite suggests that it may require more aggressive conditions for dissolution, and its low dissolution under these conditions is consistent with its mineralogical stability.

Figures S4 a and b present the examples of backscattered electron (BSE) of the residue in HCl and citric acid systems, respectively. As shown, cracked and rough surface textures are observed on the particles.

**Fig. 9:**
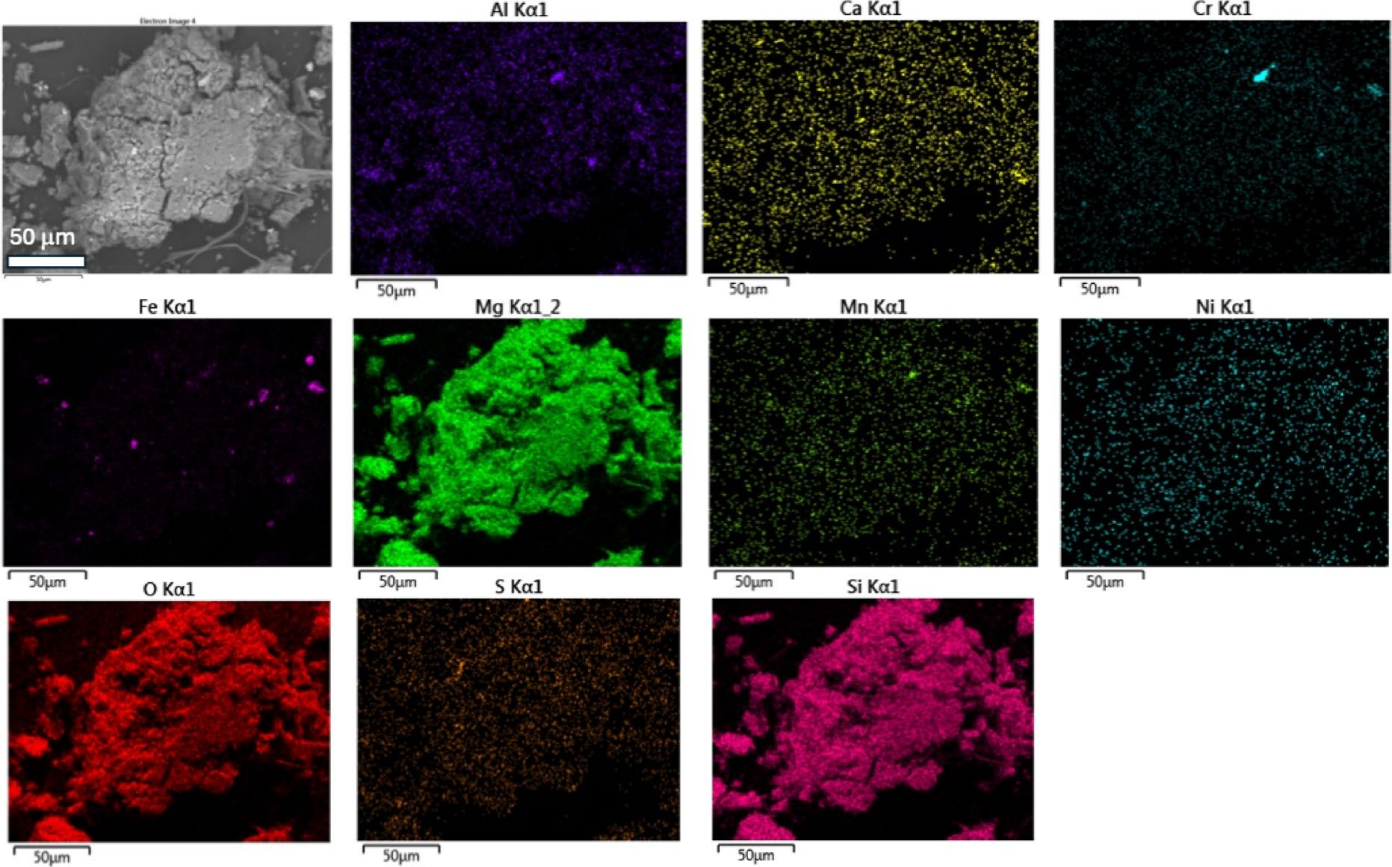
BSE image and elemental mapping of the leaching residue in HCl system at 1 mol L^- 1^ acid concentration, 25 °C, S/L ratio of 0.25 g mL-1 and agitation rate of 400 rpm during 180-min residence time.

**Fig. 10:**
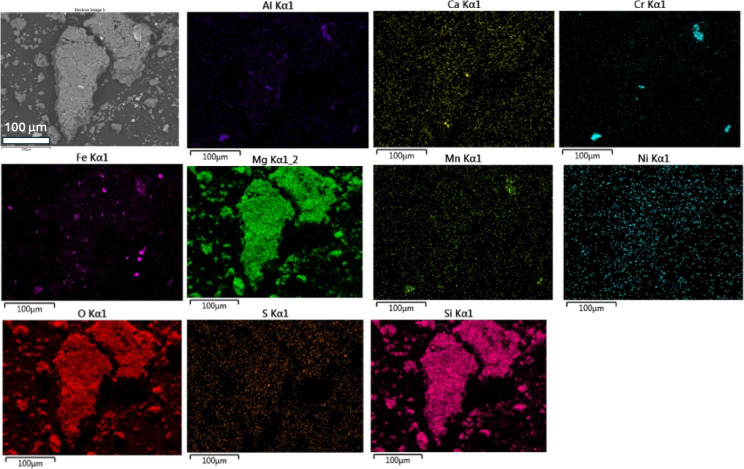
BSE image and elemental mapping of the leaching residue in citric acid system at 1 mol L^- 1^ acid concentration, 25 °C, S/L ratio of 0.25 g mL^- 1^ and agitation rate of 400 rpm during 300-min residence time.

Figures 9 and 10 present the elemental mapping of the residue in HCl and citric acid systems, respectively. As shown, Mg, Si and O are still the dominating elements which are attributed to Lizardite-1T. Particles containing Fe, Mg, and O are also detected. The concentration of Al, Ni, and Mn is low in the samples and because of that their phases were not detected by XRD.

**Fig. 11:**
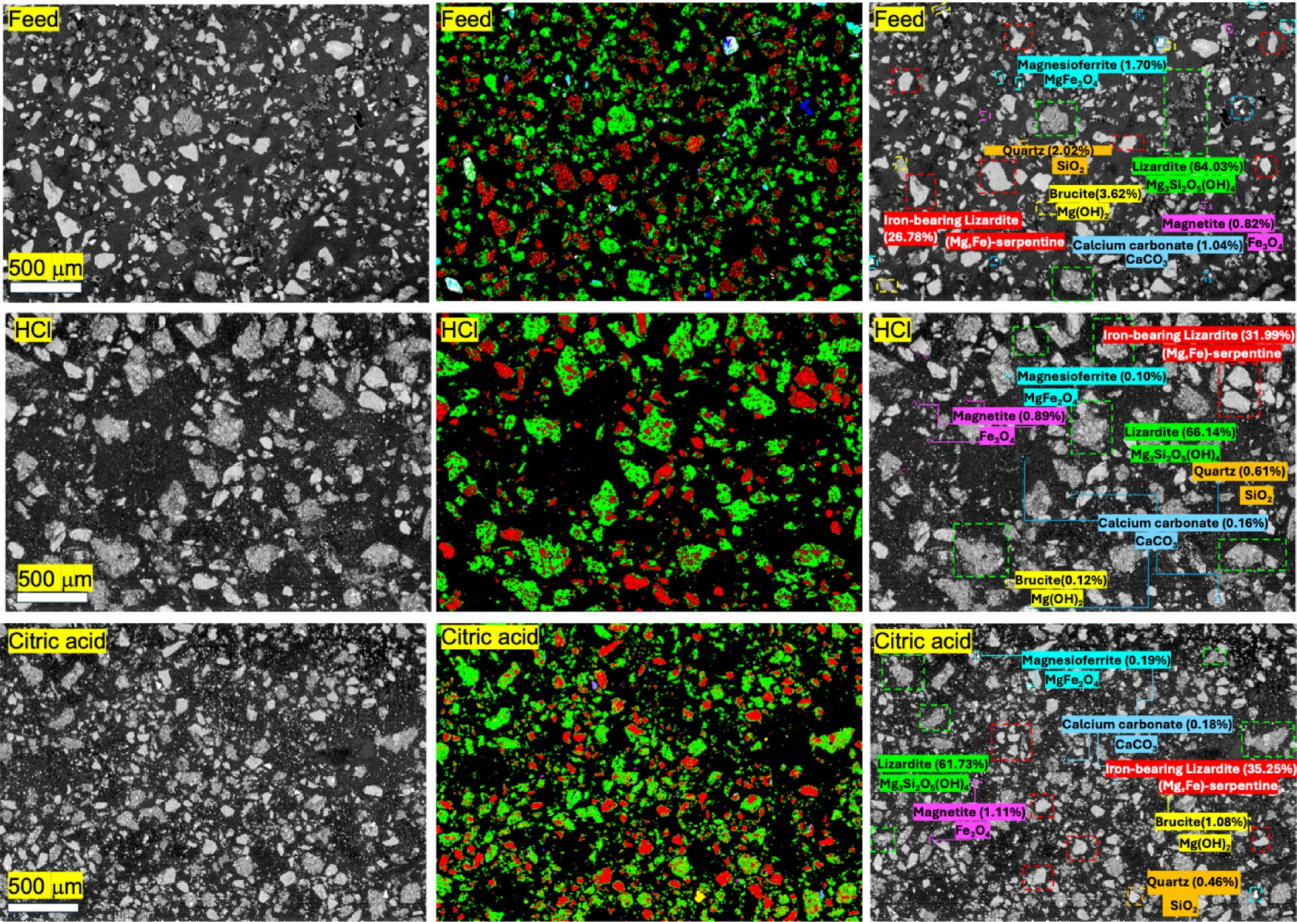
BSE image and EPMA phase mapping of the feed and leaching residues in HCl and citric acid systems, conducted at an acid concentration of 1 mol L^- 1^, 25 °C, solid-to-liquid (S/L) ratio of 0.25 g mL^- 1^, and agitation rate of 400 rpm over a 300-minute residence time. Phases are color-coded as follows: brucite (gray), calcium carbonate (blue), magnesioferrite (cyan), magnetite (magenta), quartz (yellow), iron-bearing lizardite (red), and lizardite (green). The normalized area percentage is also shown.

Figure S5-S7 present the EPMA elemental mapping, including the back-scattered electron (BSE) images of the feed and residue in HCl and citric acid systems, respectively. The elemental mapping obtained with SEM-EDX was focused on single particles. To provide elemental mapping over a wider region of the sample and obtain phase mapping, EPMA analysis was conducted. As shown, the EPMA results corroborate the findings from Rietveld analysis of the mineral phases and SEM analysis, showing high concentrations of Mg, O, and Si across all three samples, attributable to the predominance of the Lizardite-1T phase, even after acid leaching. Figure 11 provides phase mapping through EPMA, including back-scattered electron (BSE) images, alongside normalized phase area percentages. Consistent with the Rietveld analysis findings, a decrease in the normalized area percentage of certain phases is evident after acid leaching. For example, the normalized area percentage of brucite declined from 3.62% in the feed to 0.12% and 1.08% after leaching with HCl and citric acid, respectively, suggesting partial dissolution of this phase. Similarly, the normalized area percentage of magnesioferrite decreased from 1.70% in the feed to 0.10% and 0.19% after leaching with HCl and citric acid, respectively. Conversely, the normalized area percentage of magnetite increased, rising from 0.82% in the feed to 0.89% and 1.11% in samples leached with HCl and citric acid, respectively. This increase in magnetite composition aligns with Rietveld analysis results, further confirming the impact of acid leaching on phase composition.

**Fig. 12:**
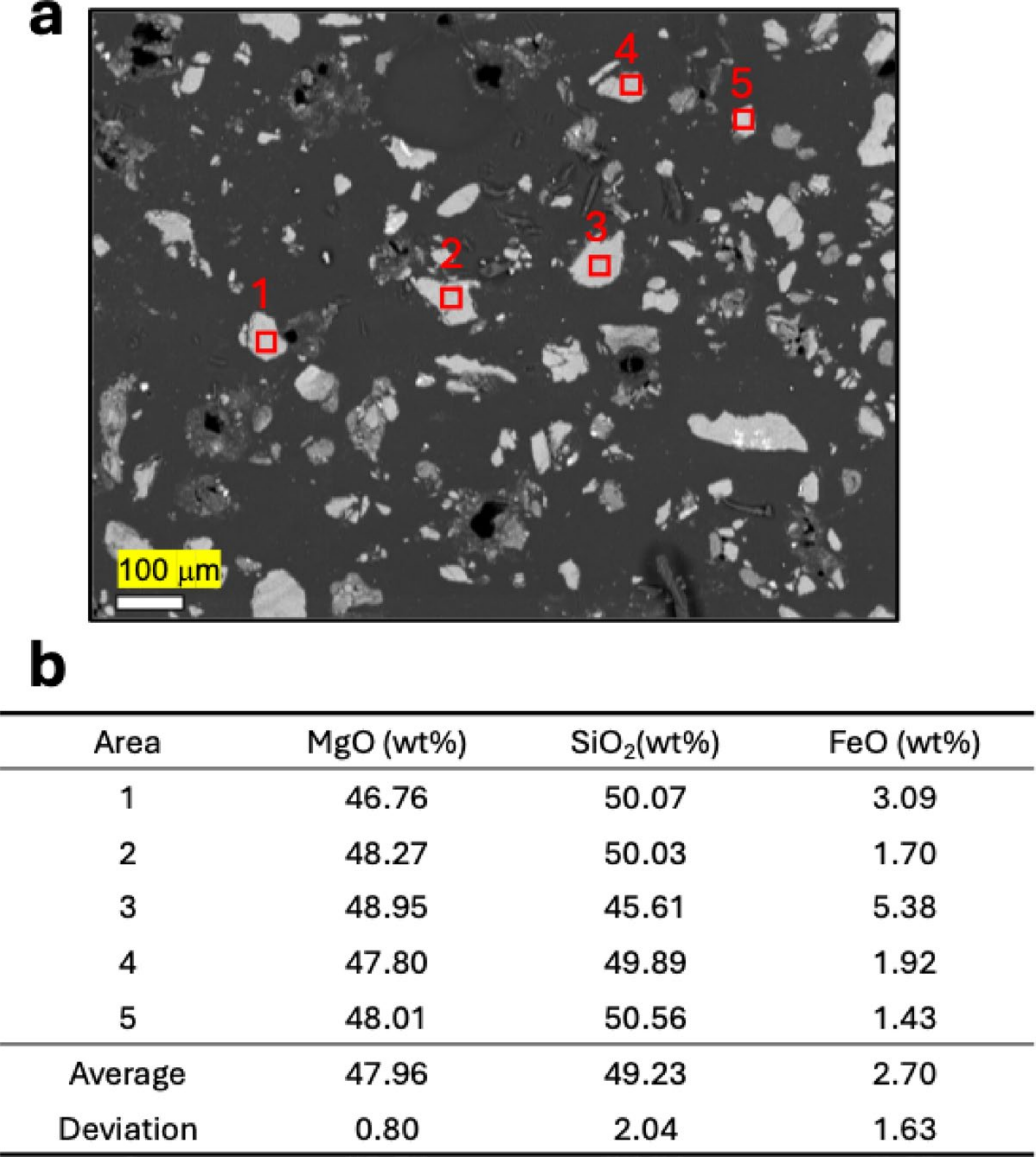
Compositional analysis of the feed sample: (**a**) backscattered electron (BSE) image highlighting five regions with elevated BSE contrast; (**b**) compositional analysis results for each of the five selected regions.

In Fig. 11, two distinct Mg-silicate phases are observed, color-coded in red and green. Among these, the green phase is more dominant, with a normalized area percentage approximately twice that of the red phase across all three samples. XRD analysis suggests that the predominant green phase corresponds to Lizardite-1T. Notably, regions with higher backscattered electron (BSE) signal intensity, or increased BSE contrast, are attributed to the alternative red Mg-silicate phase. Compositional analysis of these high-contrast regions in Fig. 12 reveals that they are primarily composed of Lizardite-1T (with a 3:2 mol ratio of MgO to SiO₂) but also contain FeO. The presence of Fe contributes to the enhanced BSE contrast, as iron-containing compounds produce stronger signals due to Fe’s higher atomic number. This suggests that the lizardite phase in the nickel tailings predominantly consists of Mg, with minor Fe substitution, forming a compositionally distinct phase within the serpentine subgroup ((Mg, Fe)₃Si₂O₅(OH)₄). Both the Mg-dominant and Fe-containing lizardite phases generally exhibit strong stability to chemical treatments with acids and thereby supporting the findings from Rietveld analysis, where these phases remained mostly unaffected.

Although direct redox measurements (e.g., Eh, Fe(II)/Fe(III) titration, or spectroscopic speciation analysis) were not performed in this study, the transformation of magnesioferrite to magnetite is supported by several independent lines of evidence. XRD results show a decrease in magnesioferrite reflections coupled with an increase in magnetite peak intensity in the post-leaching residues. BSE/EPMA imaging similarly reveals partial dissolution of Fe-bearing spinel phases and the development of magnetite-rich domains. These observations provide consistent mechanistic support for the proposed magnesioferrite to magnetite transformation. Future work will incorporate direct redox measurements to more fully quantify Fe speciation during leaching.

#### Detailed chemical reactions and thermodynamic considerations

To further clarify the leaching mechanisms in both the HCl and citric acid systems, the principal dissolution, redox, and complexation reactions were compiled together with their corresponding equilibrium constants at 25 °C (Table 5). These reactions help explain the differential reactivity of brucite, serpentine (lizardite), and iron-bearing spinel phases under the conditions examined.

**Table 5:** Key dissolution, complexation, and redox reactions relevant to acid leaching of ultramafic nickel tailings (25 °C).

Reaction Type	Reaction	Equilibrium Constant (K or log K)	Notes
Brucite dissolution	Mg(OH)₂(s) + 2 H⁺ ⇌ Mg²⁺ + 2 H₂O	K ≈ 10³⁴	Highly favorable; explains extensive brucite dissolution.
Lizardite dissolution	Mg₃Si₂O₅(OH)₄(s) + 6 H⁺ ⇌ 3Mg²⁺ + 2SiO₂·H₂O(s) + 3 H₂O	K ≈ 10⁻⁷–10⁻⁹	Thermodynamically unfavorable; explains serpentine persistence.
Magnesioferrite dissolution	MgFe₂O₄(s) + 8 H⁺ ⇌ Mg²⁺ + 2Fe³⁺ + 4 H₂O	log K ≈ − 5	Releases Fe for subsequent redox reactions.
Magnetite dissolution	Fe₃O₄(s) + 8 H⁺ ⇌ Fe²⁺ + 2Fe³⁺ + 4 H₂O	log K ≈ − 5	Occurs simultaneously with reprecipitation pathways.
Magnetite formation (redox)	2Fe³⁺ + Fe²⁺ +4O^2−^ ⇌ Fe₃O₄(s)	log K ≈ 15	Explains observed increase in magnetite in residues.
Fe(III) hydrolysis	Fe³⁺ + 3 H₂O ⇌ Fe(OH)₃(s) + 3 H⁺	log K ≈ − 38	Suppressed at low pH; magnetite formation preferred.
Mg–citrate complexation	Mg²⁺ + HCit²⁻ ⇌ MgHCit⁺	log K ≈ 2.5	Supports continued Mg dissolution in citric acid.
Ni–citrate complexation	Ni²⁺ + HCit²⁻ ⇌ NiHCit⁺	log K ≈ 4–5	Enhances Ni mobility and extraction.
Fe–citrate complexation	Fe³⁺ + Cit³⁻ ⇌ FeCit	log K ≈ 11–12	Strongest complex in the system; stabilizes Fe in solution.
Ca–citrate complexation	Ca²⁺ + HCit²⁻ ⇌ CaHCit⁺	log K ≈ 3.2	Prevents CaCO₃ precipitation during leaching.

To further elucidate the leaching mechanisms, the principal dissolution reactions for brucite and serpentine minerals under acidic conditions are provided below. Brucite dissolution in hydrochloric acid proceeds according to:

1 $$Mg\left( {OH} \right)_2\left( s \right){\rm{ }} + {\rm{ }}2{H^ + }\:{\rightleftharpoons}\:Mg{^ + }{\rm{ }} + {\rm{ }}2HO\quad \quad K{\rm{ }} \approx {\rm{ }}10\;\;\;\;\;$$

Similarly, serpentine (lizardite) dissolution can be represented as:

2 $$M{g_3}S{i_2}{O_5}\left( {OH}\right)_4\left( s \right){\rm{ }} + {\rm{ }}6{H^ + }\:{\rightleftharpoons}\:3Mg{^ {2+} }{\rm{ }} + {\rm{ }}2SiO_2.{H_2}O\left( s \right){\rm{ }} + {\rm{ }}3{H_2}O\quad \quad K{\rm{ }} \approx {\rm{ }}{10^{ - 7}}-{10^{ - 9}}$$

The significantly higher equilibrium constant for brucite dissolution indicates its much greater susceptibility to proton-promoted dissolution, consistent with the experimental observation of partial brucite removal and limited serpentine decomposition.

For citric acid, metal extraction involves both proton-promoted dissolution and complexation. The primary Mg-citrate complexation reaction is:

3 $$Mg{^{2+} }{\rm{ }} + {\rm{ }}HCit{^{2-}}\:{\rightleftharpoons}\:MgHCi{t^ + }\quad \quad {\mathrm{log}}\:\:{\rm{ }}K{\rm{ }} \approx {\rm{ }}2.5$$

This moderate stability constant explains why citric acid achieves comparable Mg extraction despite being a weak acid, as complexation shifts the equilibrium toward continued dissolution. Comparable reactions apply for Fe³⁺ and Ni²⁺, which form stronger citrate complexes (log K values typically in the range 4–11). These thermodynamic relationships explain the experimentally observed behavior: (i) extensive brucite dissolution, (ii) partial dissolution of magnesioferrite, (iii) re-precipitation and growth of magnetite under acidic conditions, and (iv) the strong persistence of lizardite, which remained largely unaffected in both leaching systems.

The partial dissolution of magnesioferrite and other Fe-bearing phases in acid is explained by the following reactions which release both Fe²⁺ and Fe³⁺ into solution:

4 $$MgFeO\left( s \right){\rm{ }} + {\rm{ }}8{H^ + }{\rm{ }} \to {\rm{ }}Mg{^ + }{\rm{ }} + {\rm{ }}2Fe{^ + }{\rm{ }} + {\rm{ }}4HO$$

5 $$FeO\left( s \right){\rm{ }} + {\rm{ }}8{H^ + }{\rm{ }} \to {\rm{ }}Fe{^ + }{\rm{ }} + {\rm{ }}2Fe{^ + }{\rm{ }} + {\rm{ }}4HO$$

Under strongly acidic conditions, Fe(III) reduction is favored (especially in the presence of organic ligands such as citric acid), suppressing Fe(OH)₃ formation and promoting re-precipitation as magnetite. This explains the observed increase in magnetite abundance in both HCl and citric acid residues.

Citric acid (H₃Cit) is a triprotic acid with pKa values pKa₁ = 3.13, pKa₂ = 4.76, and pKa₃ = 6.40. During leaching, solution pH increased from ~ 1.3 to ~ 3.6. The dominant citrate species change accordingly:

pH < 2: H₃Cit (fully protonated) + minor H₂Cit⁻.

pH 2–4: H₂Cit⁻ and HCit²⁻ become dominant.

pH > 4: Cit³⁻ appears progressively.

The ligand HCit²⁻ is the most important species for metal complexation in the pH range studied. Mg, Ni, Ca, and Mn form moderate-strength citrate complexes that pull dissolution equilibria forward, enabling extraction even when citric acid is weaker than HCl. Fe(III) forms extremely stable citrate complexes (log K ≈ 11–12), allowing Fe to remain soluble and preventing Fe(OH)₃ precipitation. With increasing temperature, both dissolution and complexation become more favorable, explaining why citric acid exhibits significantly improved leaching performance at elevated temperatures.

### Implications of co-leached species for carbonation

Although the primary objective of the leaching step is to extract Mg (and Ca), several impurity metals, including Ni, Mn, Fe, and trace amounts of Si and S, were also mobilized into solution. These species can influence carbonation behavior through competitive precipitation or complexation, depending on their speciation during the pH-swing step.

#### Fe and Mn

Upon increasing pH during carbonation, Fe³⁺ and Mn²⁺ readily form hydroxides or mixed metal hydroxides due to their low solubility at alkaline pH. Their precipitation occurs at lower pH values than Mg carbonation, meaning that Fe and Mn removal does not significantly compete with MgCO₃ or CaCO₃ precipitation. Instead, their early precipitation may actually reduce impurity incorporation into Mg carbonates and thereby improve carbonate purity.

#### Ni

Nickel remains predominantly in the soluble Ni²⁺ form throughout most of the carbonation window (pH 7–10). Because Ni-carbonate phases are less thermodynamically favored and nucleate more slowly than Mg/Ca carbonates, Ni is unlikely to interfere with the main carbonation reaction. This creates an opportunity for selective Ni recovery after carbonation, which could improve overall process economics and align with critical-metal recovery strategies.

#### Sulfate (S)

Sulfate ions do not significantly interact with Mg-carbonate formation at the concentrations observed in the leachates. However, in Ca-rich systems, high sulfate loading may promote CaSO₄ precipitation rather than CaCO₃ formation during carbonation. In the present study, sulfate concentrations were sufficiently low that gypsum formation during carbonation was not detected.

The presence of dissolved Ni, Fe, and Mn creates potential for a process integration strategy in which these metals are selectively precipitated or extracted prior to carbonation. Fe and Mn can be removed via controlled pH adjustment to form hydroxides, while Ni can be recovered downstream via hydroxide precipitation, sulfide precipitation, or solvent extraction. Such recovery steps may improve leachate purity, enhance carbonation efficiency by reducing competing reactions, and provide additional economic benefit by valorizing co-leached metals. These possibilities provide promising avenues for future process optimization.

### Integration with indirect carbonation flowsheet

#### Influence of chloride and citrate complexes on carbonation availability

In both acid systems, metal–ligand interactions influence aqueous speciation at low pH but do not prevent carbonate formation during the subsequent carbonation step. In the HCl leachates, Mg²⁺, Ca²⁺, and Mn²⁺ form only weak outer-sphere chloride complexes (e.g., MgCl⁺), which readily dissociate as the pH increases during carbonation. Consequently, chloride does not impede the availability of free divalent cations for MgCO₃ or CaCO₃ precipitation.

In the citric acid system, Mg²⁺, Fe³⁺, and Ni²⁺ form stable aqueous citrate complexes at low pH. However, these complexes are pH-sensitive, and the increase to carbonation conditions (pH 8–10) shifts the equilibrium toward ligand deprotonation and metal release. At elevated pH, Fe and Mn preferentially precipitate as hydroxides, whereas Mg remains soluble until supersaturation with respect to magnesium carbonate is reached. Thus, ligand-promoted dissolution in the leaching step does not inhibit Mg availability for subsequent carbonation.

#### Integration of acid leaching into an indirect CO₂ mineralization flowsheet

The acid leaching approach used here is designed as the first stage of a pH-swing indirect mineralization process. A practical flowsheet includes: (i) Acid leaching (pH 0–2): dissolution of Mg and Ca from ultramafic tailings using either HCl or citric acid; metal–chloride and metal–citrate complexes remain fully soluble at this stage. (ii) Optional impurity removal: Fe and Mn can be removed by controlled pH elevation (pH 4–6), while Ni can be selectively recovered downstream, improving carbonate purity and process economics. (iii) Carbonation step (pH 8–10): pH is raised using alkaline reagents (e.g., NaOH, Ca(OH)₂, NH₃), releasing complexed metals, inducing supersaturation, and promoting the precipitation of MgCO₃, CaCO₃, or hydrated magnesium carbonates.

This sequence ensures that the acid leaching step is fully compatible with industrial indirect mineralization processes, and that metal–ligand complexes formed during leaching do not hinder the availability of divalent metal ions required for CO₂ sequestration.

### Thermodynamic basis for acid selection: insights from OLI modeling

To support the selection of leaching reagents used in this study, comprehensive thermodynamic simulations were conducted using OLI Studio (v. 12.0) to evaluate the dissolution behavior of ultramafic tailings in the presence of various inorganic (HCl, H₂SO₄, HNO₃) and organic (citric, acetic, and oxalic) acids. These simulations provided a predictive assessment of mineral dissolution, secondary phase formation, and aqueous speciation over a wide pH range. The goal was to identify acids that (i) maximize Mg extraction, (ii) avoid the formation of sparingly soluble precipitates that could inhibit leaching, and (iii) remain compatible with downstream CO₂ mineralization.

#### Inorganic acids: comparison of HCl, H₂SO₄, and HNO₃

**Fig. 13:**
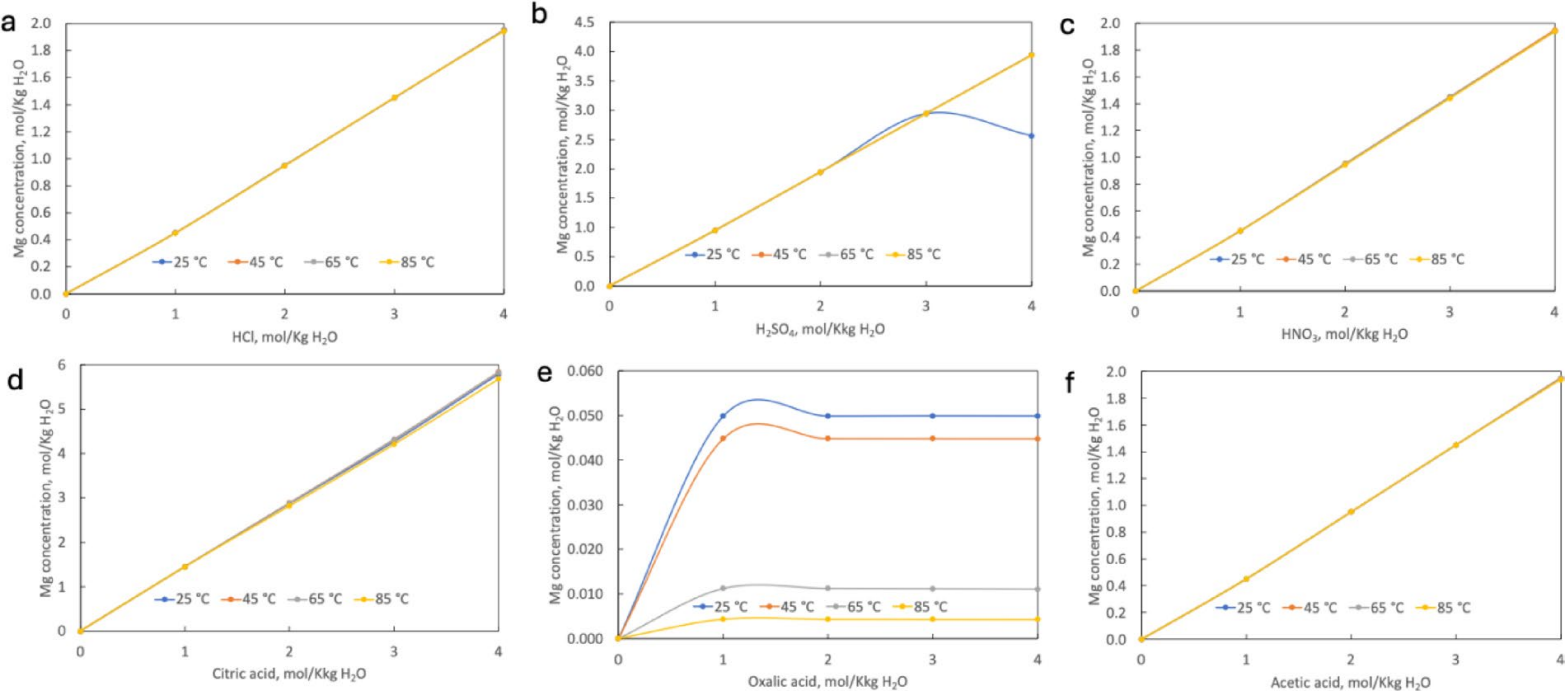
OLI thermodynamic predictions showing the effect of acid type and temperature (25–85 °C) on Mg dissolution from ultramafic tailings. (**a**) HCl, (**b**) H₂SO₄, and (**c**) HNO₃ represent strong inorganic acids, while (**d**) citric acid, (**e**) oxalic acid, and (**f**) acetic acid represent organic acids with distinct dissolution mechanisms.

**Fig. 14:**
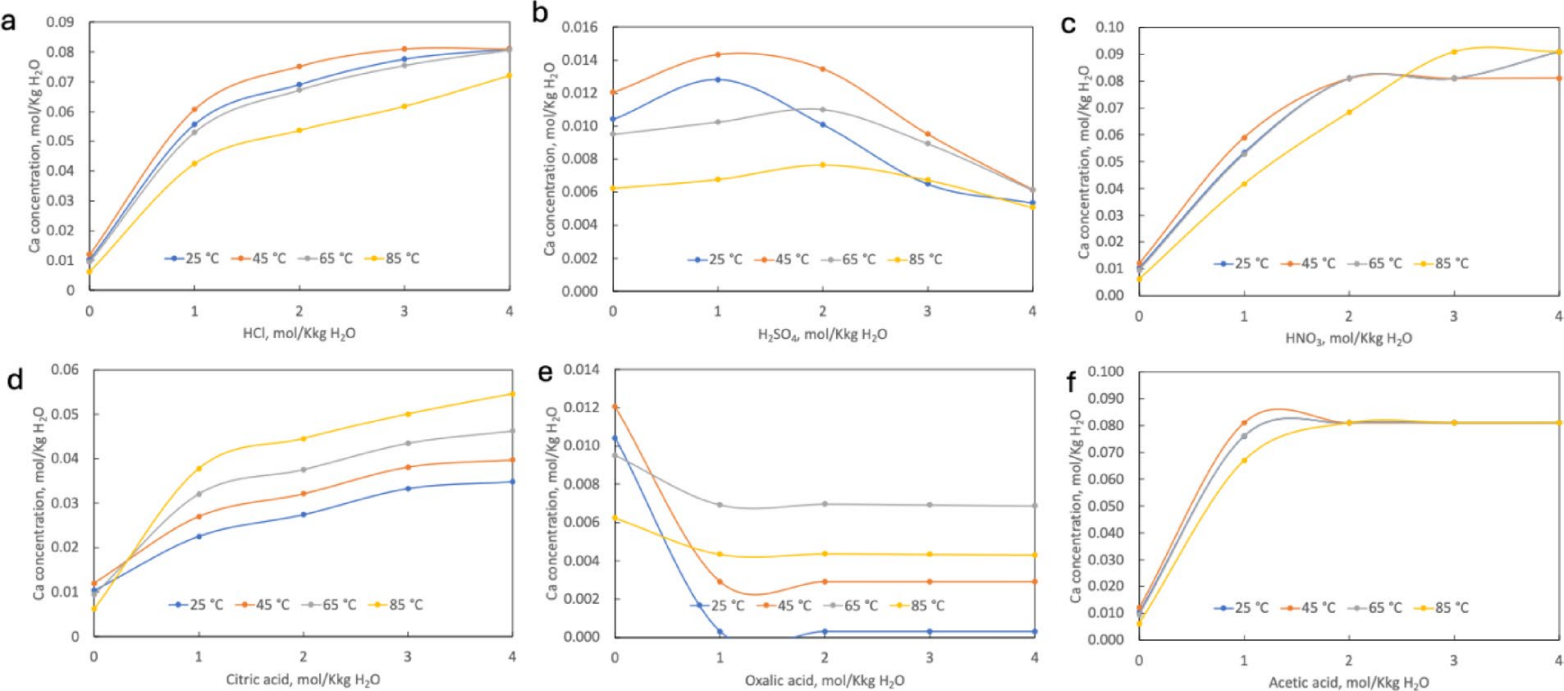
OLI thermodynamic predictions showing the effect of acid type and temperature (25–85 °C) on Ca dissolution from ultramafic tailings. (**a**) HCl, (**b**) H₂SO₄, and (**c**) HNO₃ represent strong inorganic acids, while (**d**) citric acid, (**e**) oxalic acid, and (**f**) acetic acid represent organic acids with distinct dissolution mechanisms.

Simulation results (Fig. 13) show that HCl and HNO_3_ provides the highest extent of Mg dissolution across the entire pH range of interest. In contrast, H₂SO₄ exhibit significantly reduced Mg solubility due to the precipitation of secondary phases. The most critical distinction arises in the Ca–H₂SO₄ system. As shown in Fig. 14, the addition of sulfuric acid leads to the rapid formation of CaSO₄·2 H₂O (gypsum) even at pH values below 2. This precipitation event has three major implications: (1) Passivation Layer Formation: Gypsum deposits onto reacting mineral surfaces, forming a low-solubility layer that inhibits the dissolution of brucite, magnesioferrite, and other reactive Mg phases. (2) Suppression of Mg Extraction: The precipitation of CaSO₄ alters solution chemistry by reducing free acid availability and increasing ionic strength, which collectively suppress Mg release. (3) Severe Operational Challenges: Gypsum scaling is a well-documented problem in acidic hydrometallurgical systems and presents major challenges in stirred reactors, piping, and heat-transfer equipment. This makes sulfuric acid unsuitable for mechanistic leaching studies where uninterrupted mineral dissolution is required.

These findings clearly show that sulfuric acid, though common in industrial leaching, is thermodynamically incompatible with ultramafic tailings due to extensive CaSO₄ precipitation. In contrast, HCl does not produce any sparingly soluble chloride phases, meaning no secondary solid phases inhibit leaching, Mg is fully mobilized into solution, Ca remains soluble at low pH, and reaction rates are not hindered by passivation. These characteristics make HCl the most appropriate inorganic acid for probing intrinsic dissolution mechanisms.

Although HNO₃ does not form CaSO₄-like precipitates, simulations predict comparable Mg solubility than HCl, potential for redox reactions with Fe-bearing phases, and higher cost and handling risks. Thus, it was considered less suitable for detailed mechanistic investigation.

#### Organic acids: citric acid versus oxalic acid and acetic acid

Thermodynamic simulations were also conducted to assess the dissolution behavior of ultramafic tailings in the presence of three representative organic acids, citric, oxalic, and acetic acid (Fig. 13). These acids were selected because they represent distinct mechanistic categories: (i) multidentate complexing agents (citric), (ii) strong precipitating ligands (oxalic), and (iii) weak monocarboxylic acids (acetic). The modeling results reveal significant differences in their ability to extract Mg and in their compatibility with subsequent CO₂ mineralization steps.

Across all temperatures examined (25–85 °C), citric acid exhibits the highest Mg dissolution capacity among the organic acids (Fig. 13). The Mg concentration increases nearly linearly with acid concentration, showing no evidence of solid precipitate formation. This favorable behavior arises from (1) Strong complexation of Mg²⁺ and Fe³⁺ by citrate species (HCit²⁻, Cit³⁻): these complexes significantly reduce the activity of free metal ions, shifting dissolution equilibria toward continued mineral breakdown. (2) Absence of sparingly soluble Mg- or Ca-citrate solids: unlike oxalic acid, citric acid does not form low-solubility metal–citrate precipitates under the modeled conditions, enabling full mobilization of dissolved species. (3) Temperature-enhanced dissolution: higher temperatures further increase Mg solubility, consistent with endothermic complexation behavior commonly reported for citrate systems.

The combination of high Mg extraction, no passivating precipitates, mild pH behavior, and environmental compatibility makes citric acid an excellent candidate for probing ligand-assisted leaching mechanisms in ultramafic tailings.

At all temperatures studied, oxalic acid exhibits very low Mg solubility, with Mg concentrations plateauing at approximately 0.04–0.05 mol/kg H₂O (Fig. 13). This is several orders of magnitude lower than for citric or acetic acid. The limited dissolution is explained by: 1) Formation of highly insoluble Mg-oxalate (MgC₂O₄·xH₂O): as soon as Mg is released into solution, it rapidly precipitates, preventing further dissolution of brucite or serpentine components.2) Precipitation of Ca-oxalate and Fe-oxalate: these solids remove Ca²⁺ and Fe³⁺ from solution, altering ionic strength and reducing acid availability for continued reaction.3) Temperature has minimal positive effect: even at 85 °C, Mg concentrations remain extremely low due to the dominant stability of oxalate precipitates.

Because oxalic acid produces extensive precipitation of metal-oxalate phases, it inhibits rather than promotes leaching, making it unsuitable for mechanistic studies or for generating Mg-rich solutions for downstream carbonation.

The Mg solubility trends for acetic acid show a nearly linear but much lower dissolution capacity compared with citric acid (Fig. 13). This behavior reflects: (1) Weak acidity (pKa ≈ 4.76): The acid has limited proton availability for mineral attack, especially for dissolving brucite and serpentine. (2) Minimal complexation with Mg²⁺: acetate forms only weak ion-pair associations with Mg, providing little thermodynamic drive for continued dissolution. (3) No precipitation issues: unlike oxalic acid, acetic acid does not form insoluble salts with Mg or Ca, but its weak acidity still limits overall extraction efficiency. Thus, while acetic acid avoids the passivation problems of oxalic acid, its low proton strength and poor complexation ability yield insufficient Mg extraction to be useful in this study.

## Conclusion

This study demonstrates that acid leaching of ultramafic nickel tailings using HCl and citric acid is an effective approach for preparing feed solutions for indirect CO₂ mineralization. HCl, due to its strong acidity and complete dissociation, consistently achieved higher extraction efficiencies for key divalent metals such as Mg and Fe. In contrast, citric acid, while exhibiting lower overall efficiency, proved advantageous in terms of environmental safety, selectivity, and lower corrosivity.

Leaching performance was strongly influenced by operational parameters. A lower solid-to-liquid ratio and moderate acid concentrations favored metal dissolution, while temperature effects varied significantly between acid systems due to differing mechanisms—protonation in HCl and complexation in citric acid. Multi-stage leaching yielded diminishing returns after the first cycle, highlighting the challenge of fully extracting metals from stable phases such as lizardite.

Mineralogical analysis confirmed partial dissolution of brucite and transformation of iron-bearing phases, while lizardite remained largely unaltered, highlighting its resistance to acid leaching under tested conditions. These findings suggest that supplementary strategies, such as thermal pre-treatment, may be required to fully exploit ultramafic tailings for CO₂ sequestration.

Overall, both acid systems offer viable pathways for metal extraction from ultramafic tailings, with the choice dependent on trade-offs between efficiency, cost, and environmental impact. The insights gained provide a foundation for optimizing leaching protocols in future mineral carbonation systems.

## Experimental

### Materials

The ultramafic nickel tailing was obtained from a nickel mine site in Ontario, Canada. The wet tailing was dried at 75 °C for four days. For the experiments, the dried tailing was sieved to obtain particles between 100 and 800 microns. A laser particle size analyzer (Malvern Mastersizer 2000) measured the particle size of the sample, revealing an average particle size of 132 microns, with d10 and d90 values of 2.58 and 453.11 microns, respectively.

Nitric acid (ACS Reagent Grade, 68.0–70.0 wt% Assay, VWR), hydrochloric acid (ACS Reagent Grade, 36.5–38% wt% Assay, VWR), and citric acid monohydrate (ACS reagent ≥ 99.0%, Milipore Sigma) were used to perform characterization and experiments. Deionized water with a resistivity of 18.2 MΩ·cm, produced by the Milli-Q Integral water purification system from MilliporeSigma (Merck KGaA, Darmstadt, Germany), was used to prepare the slurry solutions. Certified multi-element standard stock solutions (Nitric background, Inorganic Ventures) were utilized for calibrating analytical instruments and conducting adsorption tests.

### Procedures

The experimental setup comprised a 1 L jacketed glass reactor equipped with a Liebig condenser, a receiving flask, and a mechanical stirrer set to 400 rpm, all controlled via a JGR-2 L, YH CHEM control panel. Vapor condensation and distilled water collection were managed using the Liebig condenser and receiving flask. To maintain a constant reactor temperature, a water bath (Fisher Scientific, Inc., ISOTEMP 4100 H21P) circulated hot water through the jacket, while a chiller (Fisher Scientific, Inc., ISOTEMP 6200 R28) circulated coolant through the condenser. This integrated equipment array ensured precise control and execution of each experimental procedure.

Experiments began by filling the reactor with the nickel tailing and acid solution at the target solid to liquid ratio. The process started by heating the bath from room temperature to the desired set temperature and maintaining this temperature throughout the experiment. Initial kinetic experiments conducted at 25 °C assessed Mg, Ca, and Ni concentrations in the solutions. Results from inductively coupled plasma optical emission spectroscopy (ICP-OES) indicated that equilibrium was reached in approximately 180 min for HCl and 300 min for citric acid. Therefore, in subsequent experiments, systems were held for 180–300 min after reaching the target temperature.

Immediately before sampling, 10 mL volumetric flasks were prepared by adding 8 mL of a 5 wt% HNO_3_ solution using the Hamilton Microlab 600 diluter/dispenser system. Samples of the saturated leach solution were withdrawn from the flasks using a syringe and plastic tubing. To remove any solid particles, nylon syringe filters (Basix, 0.45 μm) were attached to the syringes, preventing any solids from re-dissolving during extraction. The filtered samples were then promptly transferred to volumetric flasks and filled up to the precisely calibrated 10 mL mark. Specifically, 2 mL samples of the saturated solution were collected for subsequent analyses.

After the experiment, the solution underwent vacuum filtration using Whatman Grade 3 filter paper, and the filtrate was collected. The filter cake was washed three times with 100 mL of deionized water. The washed filter cake was then dried in an oven at 50 °C for 24 h. Once fully dried, it was placed into sample bags and stored in a desiccator until analysis.

The leaching efficiency was calculated using the following equation:

6 $$\:Leaching\:efficiency\:\left(\mathrm{\%}\right)=\frac{{V}_{leachate}\times\:{C}_{i,leachate}}{{m}_{feed}\times\:{C}_{i,feed}}\times\:100$$

where V_leachate_ is the volume of leachate (L), C_i, leachate_ is the concentration of element i in the leachate (mg L^− 1^), m_feed_ is the mass of Ni tailing feed (kg), C_i, feed_ is the concentration of element i in the solid feed (mg L^− 1^).

### Characterization

#### Compositional analysis

The Ni tailing sample was analyzed using lithium metaborate and tetraborate fusion followed by ICP-OES. Magnesium (Mg), calcium (Ca), manganese (Mn), aluminum (Al), iron (Fe), nickel (Ni), chromium (Cr), sulfur (S), and silicon (Si) concentrations were measured using ICP-OES with a PerkinElmer Optima 8000 instrument at wavelengths of 280.271 nm for Mg, 422.673 nm for Ca, 257.610 nm for Mn, 396.153 nm for Al, 238.204 nm for Fe, 231.604 nm for Ni, 267.716 nm for Cr, 181.975 nm for S, and 251.611 nm for Si. The ICP-OES system was calibrated using standards with concentrations ranging from 1 to 50 mg/L, prepared from certified standard solutions from Inorganic Ventures. The uncertainty of ICP-OES measurements was assessed through repeated calibration and testing with standard solutions, resulting in an average relative standard deviation of 5% for Mg, Ca, Mn, Al, Fe, Ni, Cr, Si and S measurements.

#### Mineralogical analysis

The sieved sample was placed in shallow well sample holders and analyzed for X-ray diffraction (XRD) using a Rigaku MiniFlex 600 Diffractometer. The analysis covered a 2θ range from 5° to 75° at a scanning rate of 1.25°/min. Rietveld analysis, with an accuracy of $\:\pm\:5\:wt\%,\:$was performed using the integrated X-ray powder diffraction software (PDXL), which adjusted a theoretical diffraction pattern to match the experimental data by refining the structural model of the material. This refinement process involved modifying parameters such as lattice constants, atomic positions, and thermal vibrations to minimize the difference between the observed and calculated diffraction patterns. The quantitative fit was assessed using a chi-squared (χ²) value, aiming for a value lower than 5. Details of the database used in the analysis are provided in Table 6.

In Rietveld analysis, the weight% (wt%) of each phase in a multi-phase sample is calculated using the refined scale factors obtained from fitting the observed diffraction pattern. The fundamental equation used is derived from the relationship between the scale factor, phase density, and unit cell volume.

7 $$\:wt{\mathrm{\%}}_{i}=\frac{\frac{{S}_{i}{Z}_{i}{M}_{i}}{{V}_{i}{\rho\:}_{i}}}{\sum\:_{j=1}^{n}\frac{{S}_{j}{Z}_{j}{M}_{j}}{{V}_{j}{\rho\:}_{j}}}\times\:100$$

where S_i_ is scale factor for phase *i* (obtained from Rietveld refinement), Z_i_ is number of formula units per unit cell for phase *i*, M_i_ is molar mass of phase *i* (g mol^− 1^), V_i_ is unit cell volume of phase *i* (Å³), ρ_i_ is density of phase *i* (g cm^−^³), and n is total number of phases in the sample.

There are a few factors to be considered for Rietveld analysis. This method cannot directly quantify amorphous phases unless an internal standard is added. If phases have significantly different X-ray absorption coefficients, absorption corrections are necessary to improve accuracy. Factors like preferred orientation, particle size, and strain can affect the scale factor and should be refined carefully.

**Table 6:** The DB card number for identified phases.

Phase name	Formula	DB card number
Lizardite-1T	Mg_3_Si_2_O_5_(OH)_4_	00–062-0393
Brucite	Mg(OH)_2_	04–013-9512
Magnetite	Fe_3_O_4_	01–088-0866
Calcium carbonate	CaCO_3_	04–012-8783
Magnesioferrite	MgFe_2_O_4_	04–012-1067

#### Morphological and elemental mapping analysis

To prepare for scanning electron microscopy with energy dispersive X-ray spectroscopy (SEM/EDX) analysis, the powder samples were mounted on carbon tape placed on SEM specimen stubs. These mounts were coated with a thin layer of carbon to reduce charging artifacts during analysis. The analyses were conducted using a Hitachi SU3500 Variable Pressure SEM, coupled with an Oxford AZtec X-Max50 SDD X-ray analyzer. EDX spectroscopy, capable of detecting elements from carbon to uranium within a few microns depth and with a detection limit of approximately 0.5 wt%, was used for semi-quantitative analysis. To examine the morphology of the samples and map the distribution of elements, an electron probe microanalyzer (EPMA) equipped with a JEOL JXA8230 5-WDS Electron Microprobe was employed. The EPMA operating conditions are outlined in Table 7. For the analysis, the samples were mixed with epoxy to form pellets without pulverization and then polished to create cross-sections for examination.

**Table 7:** Operating conditions used in EPMA.

Parameters	Value
Accelerating voltage	10.0 kV
Probe current	30 nA
Mode	SPOT
Probe diameter (nom)	1 μm
Dwell time	10.00 ms
Direction	Single
Points	750$\:\times\:$535
Interval	X: 2.00 Y:2.00

### Statistical analysis

To ensure reliability and assess the precision of the experimental data, each test was conducted in triplicate (*n* = 3). For each condition, the arithmetic mean (µ) and standard deviation (σ) were calculated using the following formulas:

8 $$\:\mu\:=\frac{1}{n}\sum\:_{i=1}^{n}{x}_{i}\:$$

9 $$\:\sigma\:=\sqrt{\frac{1}{n-1}\sum\:_{i=1}^{n}({x}_{i}-\mu\:{)}^{2}}$$

The Relative Standard Deviation (RSD), expressed as a percentage, was used to evaluate the precision of the measurements and calculated as:

10 $$\:RSD\left(\mathrm{\%}\right)=\frac{\sigma\:}{\mu\:}\times\:100$$

Error bars representing ± 1 standard deviation (σ) were plotted on all relevant graphs to visually represent the variability among replicates. This approach allows for clear assessment of data consistency and experimental reproducibility and facilitates comparison across different experimental conditions.

## Supplementary Information

Below is the link to the electronic supplementary material.

Supplementary Material 1

## Data Availability

The data will be made available upon request. Please contact the corresponding author, Dr. Gisele Azimi (g.azimi@utoronto.ca).
