# Supplementary material for: Acid leaching process of an ultramafic mine tailing for indirect CO2 mineralization
Source: Sci Rep. 2026 Mar 14;16:21020. doi: 10.1038/s41598-026-35873-z (PMC13342521; doi:10.1038/s41598-026-35873-z)
Supplement: Supplementary file 1 — Supplementary Material 1 [file 41598_2026_35873_MOESM1_ESM.docx]

**Supplementary Materials**

**Acid Leaching Process of an Ultramafic Mine Tailing for Indirect CO_2_ Mineralization**

Kyoung Hun Choi^a^, Spencer Cunningham^a^, Hamid Radfarnia^b^, Kourosh Zanganeh^b^, Gisele Azimi^a,*^

^a^ Laboratory for Strategic Materials, Department of Chemical Engineering and Applied Chemistry, University of Toronto, 200 Collge St., Toronto, ON, M5S3E5, Canada.

^b^ Natural Resources Canada, CanmetENERGY-Ottawa, 1 Haanel Drive, Ottawa, ON, K1A1M19, Canada.

*Corresponding Author Email: [g.azimi@utoronto.ca](mailto:g.azimi@utoronto.ca)

Number of Pages: 5

Number of Figures: 7

**List of Figures**

Figure S1. a) Contribution of various sectors to CO_2_ emissions globally. b) Share of major contributing countries ^1^.

Figure S2. Secondary electron (SEM) image of the feed Ni tailing sample.

Figure S3. Kinetic experimental results for the concentration of a) Mg, b) Ni, and c) Ca at 25 °C, 1 mol L^-1^ acid concentration, S/L of 0.25 g mL^-1^, and agitation rate of 400 rpm, up to 360 min residence time. The error bars are determined based on three replicates.

Figure S4. BSE images of the leaching residue in a) HCl, b) citric acid at 1 mol L^-1^ acid concentration, 25 °C, S/L ratio of 0.25 g mL^-1^ and agitation rate of 400 rpm during 180- and 300-min residence time, respectively.

Figure S5. BSE image and EPMA elemental mapping of the feed Ni tailing sample.

Figure S6. BSE image and EPMA elemental mapping of the leaching residue in HCl system at 1 mol L^-1^ acid concentration, 25 °C, S/L ratio of 0.25 g mL^-1^ and agitation rate of 400 rpm during 180-min residence time.

Figure S7. BSE image and EPMA elemental mapping of the leaching residue in citric acid system at 1 mol L^-1^ acid concentration, 25 °C, S/L ratio of 0.25 g mL^-1^ and agitation rate of 400 rpm during 300-min residence time.

Figure S1. a) Contribution of various sectors to CO_2_ emissions globally. b) Share of major contributing countries ^1^.

Figure S2. Secondary electron (SEM) image of the feed Ni tailing sample.

Figure S3. Kinetic experimental results for the concentration of a) Mg, b) Ni, and c) Ca at 25 °C, 1 mol L^-1^ acid concentration, S/L of 0.25 g mL^-1^, and agitation rate of 400 rpm, up to 360 min residence time. The error bars are determined based on three replicates.

Figure S4. BSE images of the leaching residue in a) HCl, b) citric acid at 1 mol L^-1^ acid concentration, 25 °C, S/L ratio of 0.25 g mL^-1^ and agitation rate of 400 rpm during 180- and 300-min residence time, respectively.

Figure S5. BSE image and EPMA elemental mapping of the feed Ni tailing sample.

Figure S6. BSE image and EPMA elemental mapping of the leaching residue in HCl system at 1 mol L^-1^ acid concentration, 25 °C, S/L ratio of 0.25 g mL^-1^ and agitation rate of 400 rpm during 180-min residence time.

Figure S7. BSE image and EPMA elemental mapping of the leaching residue in citric acid system at 1 mol L^-1^ acid concentration, 25 °C, S/L ratio of 0.25 g mL^-1^ and agitation rate of 400 rpm during 300-min residence time.

**References**

1. Neeraj & Yadav, S. Carbon storage by mineral carbonation and industrial applications of CO2. *Mater. Sci. Energy Technol.* **3**, 494–500 (2020).
